# From inserts to chips: microfluidic culture and 3D astrocyte co-culture drive functional and transcriptomic changes in hiPSC-derived endothelial cells

**DOI:** 10.1186/s12987-025-00672-7

**Published:** 2025-06-16

**Authors:** Tuuli-Maria Sonninen, Sanni Peltonen, Sara Kälvälä, Hoang-Tuan Nguyen, Marika Ruponen, Prateek Singh, Šárka Lehtonen

**Affiliations:** 1 A.I. Virtanen Institute, University of Eastern Finland, Kuopio, Finland; 2 Finnadvance, Helsinki, Finland; 3 School of Pharmacy, Faculty of Health Sciences, University of Eastern Finland, Kuopio, Finland

**Keywords:** BBB, Microfluidic chip, HiPSC, Endothelial cell, Astrocyte, Co-culture, Single-cell RNA sequencing

## Abstract

**Background:**

The blood-brain barrier (BBB) exhibits a hurdle for drug delivery and development. In addition, the dysfunction of the BBB has been seen in several neurodegenerative diseases, although the mechanisms remain poorly understood. Thus, improved models are needed for the purposes of disease modelling and drug development. To overcome the constraints of conventional in vitro models, there has been a growing use of human induced pluripotent stem cells (hiPSCs) and organ-on-chip systems. However, the detailed characterization of these models is still mainly missing. We aimed to investigate how different culture platforms alter the functionality and, consequently, the transcriptomic phenotype of hiPSC-derived endothelial cells (ECs).

**Methods:**

ECs were cultured on a microfluidic BBB chip platform (AKITA plate) or a standard cell culture insert model. Furthermore, we used hiPSC-derived astrocytes in the AKITA plate format to examine their effect on ECs. Astrocytes were cultured under either 2D or 3D conditions. The impact of pore size and culture system was studied using permeability assays and protein expression. Finally, we used single-cell RNA sequencing to analyze transcriptional changes in ECs cultured on insert or AKITA plate, both with and without astrocytes.

**Results:**

First, we tested the impact of different membrane pore sizes in AKITA plate on EC morphology and barrier formation. We demonstrated that the AKITA plate supports confluent monolayer formation, even with higher pore sizes. Secondly, ECs cultured on AKITA plate showed improved barrier function and reduced migration in comparison to ECs cultured on inserts, supported by permeability experiments and transcriptomics. The single-cell RNA sequencing revealed the activation of cholesterol metabolism-related pathways in ECs cultured on an AKITA plate under flow conditions. At last, we discovered that astrocytes require 3D culture to sustain the EC monolayer. Moreover, astrocytes promote a slight shift in transcription levels by upregulating genes associated with EC-astrocyte interactions.

**Conclusions:**

Complex cell culture systems are becoming accessible; still, additional research into their properties is needed. Our data highlights the importance of the cell environment and its impact on the cellular function and gene expression profiles. Understanding these changes can improve future models and facilitate the development of more physiologically relevant platforms.

**Supplementary Information:**

The online version contains supplementary material available at 10.1186/s12987-025-00672-7.

## Introduction

Blood-brain barrier (BBB) is a specific functional structure that separates the brain from the bloodstream [1]. Endothelial cells (ECs) are the core cell type of BBB, and they form vessels in the brain. The properties of brain ECs are maintained by surrounding cells, including pericytes and astrocytes. Brain ECs differ from peripheral ECs; for example, they highly express tight junctions and transporter systems. Astrocytes are connected to the basement membrane of the vasculature with their polarized endfeet and support the barrier properties by releasing supporting factors [2, 3]. Pericytes enwrap the vessels and are essential for the formation and maintenance of the BBB [4]. The BBB performs multiple roles, including the delivery of oxygen and nutrients to the brain, the removal of toxic waste products, the protection of the brain from harmful substances, and limitation of bacteria, virus or cell extravasation [5]. Overall, the proper function of the BBB is crucial for brain health. The restrictive nature of the BBB prevents most of the drugs from entering the brain [6]. The dysfunction of the BBB has been recognized in several neurological disorders, including Alzheimer´s and Parkinson´s diseases [7]. Thus, there´s a need for human-based models for drug permeation studies as well as for disease modelling.

Traditionally, BBB modelling is relied on immortalized cell lines or primary cells grown in 2D conditions. Although primary cells offer numerous advantages, acquiring them from the brain tissue is limited [8]. Also, these cells tend to lose their phenotype after a few passages. Immortalized cell lines serve as an alternative to primary cells. Although they are easy to obtain and can be passaged several times, their barrier properties are typically insufficient [9]. To address the limitations of primary cells and cell lines, human induced pluripotent stem cells (hiPSCs) are increasingly being used. HiPSCs can be expanded in vitro, differentiated into every type of cell in the body, and used to build isogenic multicellular models [10, 11]. Because hiPSCs carry genetic information from the donor, one major advantage is that they can be used to investigate disease mechanisms related to risk or disease-causing genes. Despite the advantages, hiPSC-derived ECs have limitations: brain like ECs present an epithelial phenotype and lack the proper response to inflammatory stimuli, whereas vascular types have vascular identity but have weaker barrier properties.

Cell culture inserts have been commonly used in the field of BBB modelling [12]. They are simple to use and get, and they allow for semi-high throughput studies and measurement of trans endothelial resistance (TEER). On the other hand, they lack flow and are impractical for complex co-culture studies. The advances in the field of microfluidic organ-on-chip have made these systems more widely available. The organ-on-chip systems typically compose of vascular and brain channels, which can be separated by a porous membrane that mimics the extracellular matrix [13]. Organ-on-chip models allow the addition of flow, and therefore, the shear stress can be induced. These models also allow for co-culture studies with several different cell types. On the other hand, organ-on-chip models require more technical knowledge, are costly, and certain models lack the ability for TEER measurement [14]. The models are still in their early stages and comprehensive characterization and validation are largely missing.

Brain EC phenotype is controlled by surrounding environment, including cellular and physical. In our study, we used hiPSC-derived vascular ECs to investigate how environmental factors alter ECs function and transcriptomics, and can these cues drive EC phenotype more towards brain like. We compared commercially available microfluidic organ-on-chip platform (AKITA plate) with a cell culture insert model. We report that culture systems have a significant impact on cell function and transcriptome at the single-cell level. Additionally, we discovered that hiPSC-derived astrocytes require 3D conditions to maintain the EC barrier and induce a small but notable shift in the transcription level.

## Materials and methods

### Cell culture

HiPSC line used for this study was acquired from TakaraBio (Y00300). The hiPSCs were cultured on Matrigel (Corning) coated dishes in E8 media (Gibco) and passaged with EDTA (Invitrogen) when 80–90% confluence was reached.

### Endothelial cell differentiation

The ECs were differentiated according to a previously published protocol with some modifications [15, 16]. To start the differentiation, hiPSCs were plated to Matrigel-coated dishes at density of 15–25 k/cm^2^ in E8 media with 5 µM of ROCK inhibitor (Selleckchem). Next day (day 1), the media was changed to Stemdiff APEL2 media (Stemcell Technologies) supplemented with 6 µM CHIR-99,021 (Axon). Cells were cultured for two days without media changes. On day 3, the media was changed to APEL2 media with an additional 25 ng/ml of BMP4, 10 ng/ml of FGF and 50 ng/ml of VEGF (all from Peprotech). Cells were cultured in this media for 3 days, changing media on day 5. On day 6, ECs were sorted by magnetic separation using CD144 microbeads according to the manufactures protocol (Milteney). CD144 positive cells were plated on Matrigel-coated dishes in Endothelial Growth Media MV2 (ECGM-MV2, PromoCell) with an additional 20 ng/ml of VEGF for one passage. ECs were expanded and cells in passage three were used for the experiments.

### Astrocyte differentiation

Astrocytes were differentiated as previously described [17]. Briefly, hiPSCs were plated on Matrigel-coated dishes in E8 with additional ROCK inhibitor. Next day, the media was changed to neural differentiation medium (NDM) supplemented with SB431542 (Sigma) and LDN-193,189 (Selleckchem) and cultured until rosettes were formed. Then, the media was replaced to NDM with additional bFGF (Peprotech) for 2 days to expand the rosettes. The rosettes were picked up and transferred to ULA-plate (Corning) in astro-differentiation media (ADM) to form spheres. The spheres were expanded in ADM supplemented with FGF and EGF (Peprotech) for 5–8 months. Before experiments, the spheres were dissociated with Accutase (StemPro, Gibco) and plated on Matrigel-coated dishes in astro-maturation medium (ADM with CNTF and BMP4 (both from Peprotech)) for five to 7 days.

### AKITA plate construction

**Fig. 1:**
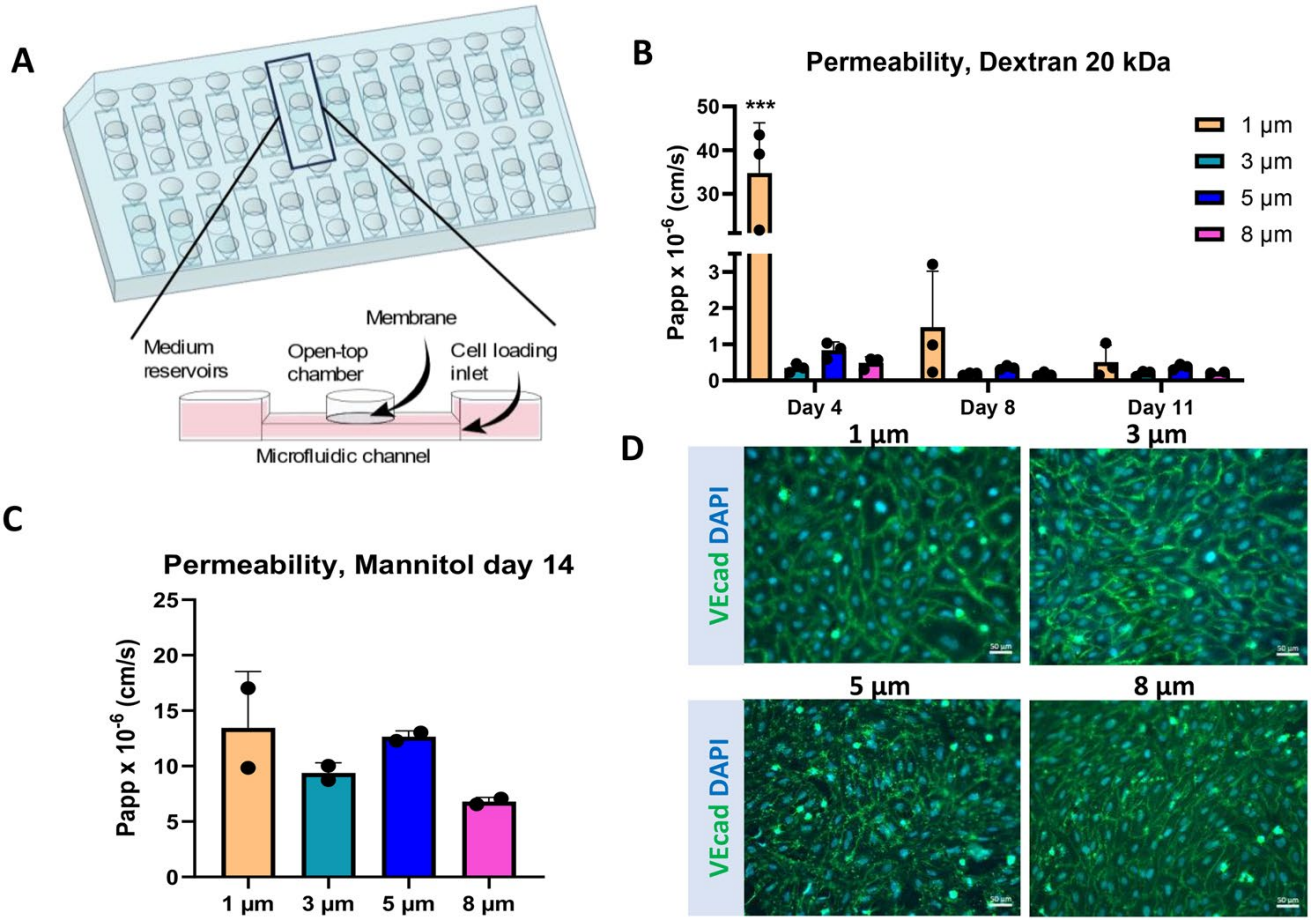
Effect of pore size on ECs cultured on AKITA plate platform (**A**) Illustration of the AKITA plate model and structure. (**B**) Permeability (Papp) of 20 kDa Dextran measured from ECs cultured on a membrane with four different pore sizes (1, 3, 5, and 8 μm). *n* = 3. Two-way Anova with Tukey´s multiple comparison tests, *** *p* < 0.001. (**C**) Permeability (Papp) of Mannitol on day 14 was measured from ECs cultured on the membrane with four different pore sizes (1, 3, 5, and 8 μm). *n* = 2. (**D**) Representative immunofluorescence images of ECs stained with VE-cadherin on day 14. ECs cultured on membranes with different pore sizes. Scale bar 50 μm. All data presented as mean +/-SD

The detailed construction of the chip is described previously [18]. AKITA Plate96-Single (AKITA plate, Finnadvance, Finland) consists of 24 (Fig. 1A) or 32 individual microfluidic units with three wells. The middle well is an open-top chamber, and the two other wells are linked together with a microchannel. The open-top chamber is separated from the microchannel by a semi-permeable polyethylene terephthalate (PET) membrane (pore size 1, 3, 5, and8 μm for this study).

### Cell loading to AKITA plate

AKITA Plate was coated with Matrigel by pipetting 50 µl of Matrigel solution to the vascular channels. The plate was incubated at 37 °C for 30 min or 4 °C overnight. For cell loading, ECs were detached with Tryple (Gibco) and counted. 2.5 million cells/ml in ECGM-MV2 were loaded onto the AKITA plate. First, the excessive volume of coating solution was removed, and 50 µl of cell suspension was added to one side of the media reservoir. Then, the plate was tilted, allowing the cell suspension to flow into the vascular channel. When cell suspension was evenly distributed, the plate was turned around and incubated for 30 min at 37 °C. Following the incubation, the plate was flipped back, and 50–100 µl of fresh ECGM MV2 was added to the media reservoirs and to open-top chambers. Cells were incubated statically overnight. Next day the plate was put on a rocker (Digital Rocker, cat# 88882002, Thermo Fisher) at settings of 10-degree tilting angle and speed of 1 RPM to induce the flow (0.6 dyne/cm^2^). For membrane pore size test, ECGM-MV2 was used throughout the culture. For rest of the assays, the media was changed to human Endothelial serum-free media (ESFM, Gibco) with additional 1x B27 (Gibco) and 10 ng/ml of FGF.

### Co-culture setup for the AKITA plate

ECs were loaded onto the AKITA plate as previously described. On the following day, matured astrocytes were added to the open-top chamber. For 2D culture, astrocytes were detached with Accutase (StemPro), and 25 × 10^3^ cells were plated in ADM on the open-top chamber. For 3D culture, Matrigel was first diluted to 5 mg/ml in ADM on ice, and 5 µl was added to the open-top chamber followed by 30 min incubation at 37 °C. During the incubation, astrocytes were detached with Accutase and counted. For one chamber, a Matrigel mixture of 120 × 10^3^ cells in 15 µl of Matrigel (5 mg/ml) was prepared on ice, and the mixture was added to the open-top chamber. Matrigel/cell mixture was incubated 30 min at 37 °C. After this, 80 µl of ADM was added to the open-top chambers, and for ECs, the media was changed to ESFM with 1x B27, 10 ng/ml FGF.

### Cell culture insert setup

ECs were plated to Matrigel-coated 24 well inserts (3 μm pore size, Sarstedt) at a density of 90 × 10^3^/cm^2^ in ECGM-MV2. The next day, the media was changed to ESFM + 1x B27 and 10 ng/ml FGF.

### Immunocytochemistry

ECs were fixed with 4% FA (VWR) for 15 min in RT or methanol (VWR) for 15 min at 4 °C followed by washes with PBS. For intracellular antigens, the ECs were permeabilized with 0.2% Triton X-100 in PBS (Sigma) for 20 min at RT. The cells were blocked with 10% horse serum (HS, Gibco) in PBS at RT for 1 h and then incubated with primary antibodies in 10% HS at 4 °C overnight. For unconjugated antibodies, the cells were incubated in 10% HS in PBS containing a secondary antibody for 1 h at RT. The cells were washed with PBS, and the nuclei were visualized with DAPI (Invitrogen). For imaging, the membranes were removed either with biopsy puncher (AKITA plate) or cutting (inserts) and mounted to glass with Fluoromount-G (Invitrogen). The cells were imaged with Zen Imager AX10. The antibodies used in this study are listed in Table 1.

**Table 1:** Antibodies used for immunocytochemistry

Type	Antibody	Origin	Vendor	Cat. No.	Dilution	Fixation
Primary	Claudin5	Mouse	Life Technologies	35-2500	1:200	MeOH
	VE cadherin	Mouse	Santa Cruz	sc-9989	1:200	FA/MeOH
	ZO1	Rabbit	Invitrogen	40-2200	1:200	FA/MeOH
	GFAP	Mouse	Sigma	MAB360	1:800	FA/MeOH
	GFAP	Rabbit	Dako	Z0334	1:500	FA/MeOH
Secondary	Anti-mouse 488	Goat	Invitrogen	A11001	1:300	
	Anti-mouse 568	Goat	Invitrogen	A11004	1:300	
	Anti-rabbit 568	Goat	Invitrogen	A11011	1:300	

### Single-cell RNA sequencing

Cell preparation was done by following the 10X cell preparation guide. Shortly, ECs grown on inserts (6 inserts) or AKITA plate (with or without astrocytes, 12 units/group) were detached with Tryple by incubating at 37°C for 10 min. After the incubation, inserts or channels were washed several times, and cells were collected to PBS with 0.04% of BSA and then passed through a 50 µm strainer (Sysmex CellTrics). Trypan blue (Sigma) was used to stain dead cells, which were excluded from the final cell count. Single cell separation was performed on the Chromium Controller platform with Chromium™ Next GEM Chip G (10x Genomics, 1000127). All samples were prepared according to the manufacturer’s instructions using the Chromium™ Next GEM Single Cell 3’ Kit v3.1 and Dual Index Kit TT Set A (10X Genomics, 1000269 and 1000215, respectively). The quality control of the library was done with a high sensitivity DNA analysis kit (Agilent, 5067 − 4626) on the 2100 Bioanalyzer instrument. Library concentration was quantified by Qubit™ dsDNA HS kit (Thermo Scientific, Q32854). Pooled libraries were sequenced on the Illumina NextSeq 500 platform using the High Output Kit v2.5 for 150 Cycles (Illumina, 20024907).

### Data analysis for the single-cell RNA sequencing

The raw data was processed on the CellRanger V7 (10X Genomics) pipeline with default parameters and mapping reads to GRCh38-2020-A transcriptome to generate count matrices. Data was analyzed with the Seurat package (version 4.3.0) on R version 4.3.2. Low-quality cells with less than 500 genes (empty droplets) or more than 10,000 genes (doublets) or cells with over 10% of reads mapping to mitochondrial genes (dying cells) were filtered out. The counts were log-normalized and top 2000 variable features were identified by vst. Dimensionality reduction (PCA) was applied, and the cells were clustered using Louvain algorithm at resolution 0.3, then visualized by uniform manifold approximation and projection (UMAP). Differentially expressed genes between all clusters were identified by Wilcoxon rank sum test (Seurat FindAllMarkers). To identify the differentially expressed genes between two clusters, Wilcoxon rank sum test (Seurat FindMarkers) was used. Genes were considered significantly differentially expressed when adjusted p-value < 0.05 and log2 Fold change > 0.5. Gene ontology enrichment and cell type analysis were done with enrichR [19–21]. Mitochondrial and ribosomal DEGs were excluded from pathway analysis. The visualization was done by using SRplot, a free online platform for data analysis and visualization [22]. Images were modified with Inkscape 1.2.

### Permeability assay

Permeability was measured with 4 and 20 kDa FITC Dextrans (Merck), Lucifer yellow (0.5 kDa, LY, Merck) and [3H] mannitol (PerkinElmer). For Dextran and LY assays on AKITA plate, media was first removed from open top chambers and media reservoirs. 100 µl of fresh media was added to the open top chambers, and 150 µl of 1 mg/ml 20 kDa dextran, 0.5 mg/ml 4 kDa dextran, or 0.1 mg/ml LY solution was added to the media reservoirs in human ESFM basal media. For inserts, 150 µl of 4 kDa dextran (0.5 mg/ml of 4 kDa) or LY (0.1 mg/ml) solution in human ESFM was added to the apical side and 800 µl of fresh media to the basolateral side. Samples (10 µl from AKITA plate and 80 µl from inserts) were collected at time points of 20, 40, 60, 90, and 120 min, and the volume was replaced with fresh media. For mannitol permeability assay, mannitol was diluted in HBSS + 25 mM HEPES, 0.004 M Sodium bicarbonate, pH 7.4 buffer at 1:1000. Channels and chambers were washed twice with HBSS buffer. After washing 250 µl of mannitol solution was added to the channels and 80 µl of HBSS buffer for the chambers. Samples were collected at time points of 10, 20, 30, 45, 60, 75, 90, and 120 min. The sample volume was replaced with HBSS buffer. Samples were collected directly to a 96-well plate (PerkinElmer) and 100 µl of Ultima Gold LSC cocktail (PerkinElmer) solution was added and left overnight at RT. The fluorescence values were measured with PerkinElmer`s VICTOR multilabel plate reader and radioactivity with MicroBeta2 2450 Microplate Counter (PerkinElmer).The background was reduced, and corrected fluorescence/CPM values were calculated. Standards were used to calculate the accumulated amounts over time. The apparent permeability coefficient (P_app_) was calculated according to the Eq. 1, where dQ/dt (µg/s) is the flux (linear range) across the barrier, A is the membrane area (cm^2^), and C_0_ is the initial concentration (µg/cm^3^).

$$\:Papp=\frac{dQ}{dt}\times\:\frac{1}{A\times\:{C}_{0}}$$

Clearance volume of empty units/inserts and cells were calculated as previously described [23, 24]. The volume cleared was plotted versus time and the slope was estimated by linear regression. The slope of the clearance curve with inserts/units and inserts/AKITA plate with cells is equal to PSf and PSt, respectively. The PS-value for ECs (PSe) was calculated. To generate the permeability coefficient, Pe, the PSe value was divided by the surface area of the filter (A in cm^2^) insert using the following equation: Pe = [1/PSt − 1/PSf]^−1^/A.

### Data analysis

The permeability data was analyzed with GraphPad Prism (version 10.0.3). Two-way ANOVA with Šídák´s multiple comparison test was used to compare ECs on insert and AKITA plate (dextran), and Tukey´s multiple comparison test for co-culture studies and membrane pore size test. For comparison of LY permeability between AKITA plate and insert, One-way ANOVA with Tukey´s multiple comparison test was used. Levels of significance *p < 0.05; **p < 0.01; ***p < 0.001 were used. Outliers were tested with the GraphPad Grubbs’ test.

## Results

### HiPSC-endothelial cells form a confluent monolayer despite larger pore sizes

In the study, we used the BBB chip model from AKITA (Fig. 1A), which allowed us to modify the construct of the membrane. Initially, we aimed to investigate whether membranes with different pore sizes impact the function and morphology of ECs when cultured on AKITA plates. We examined the impact of four different pore sizes, ranging from 1 to 8 μm, on the permeability of ECs (Fig. 1B, C). ECs cultured on 1 μm pore size membranes showed a significantly higher 20 kDa Dextran permeability on day 4 than on days 8 and 11 (Fig. 1B). No significant differences were seen among the ECs cultured on 3, 5, or 8 μm pore size membranes. We also assessed the permeability of mannitol on day 14. ECs cultured on membranes with 3 and 8 μm pore size showed slightly lower permeability in comparison to those with 1 and 5 μm (Fig. 1C). Next, we evaluated the effect of pore sizes on the expression of adherent junction protein VE-cadherin (Fig. 1D). Although VE-cadherin was expressed in all conditions, ECs cultured on 5 μm pore size showed less continuous staining, whilst those cultured on 1 μm displayed a more enlarged morphology. Furthermore, images taken from ECs stained with CD31 from both sides of the membrane demonstrate that no migration was observed, even with larger pore sizes (Fig. S1). Based on these findings, the 3 μm and 8 μm pore sizes optimally supported the EC functionality. Due to the decreased permeability shown by ECs on 3 μm pore size membranes as early as 4 days, this membrane was selected for the following studies.

### HiPSC-endothelial cells exhibit more tighter barrier on AKITA plate compared to standard insert model

**Fig. 2:**
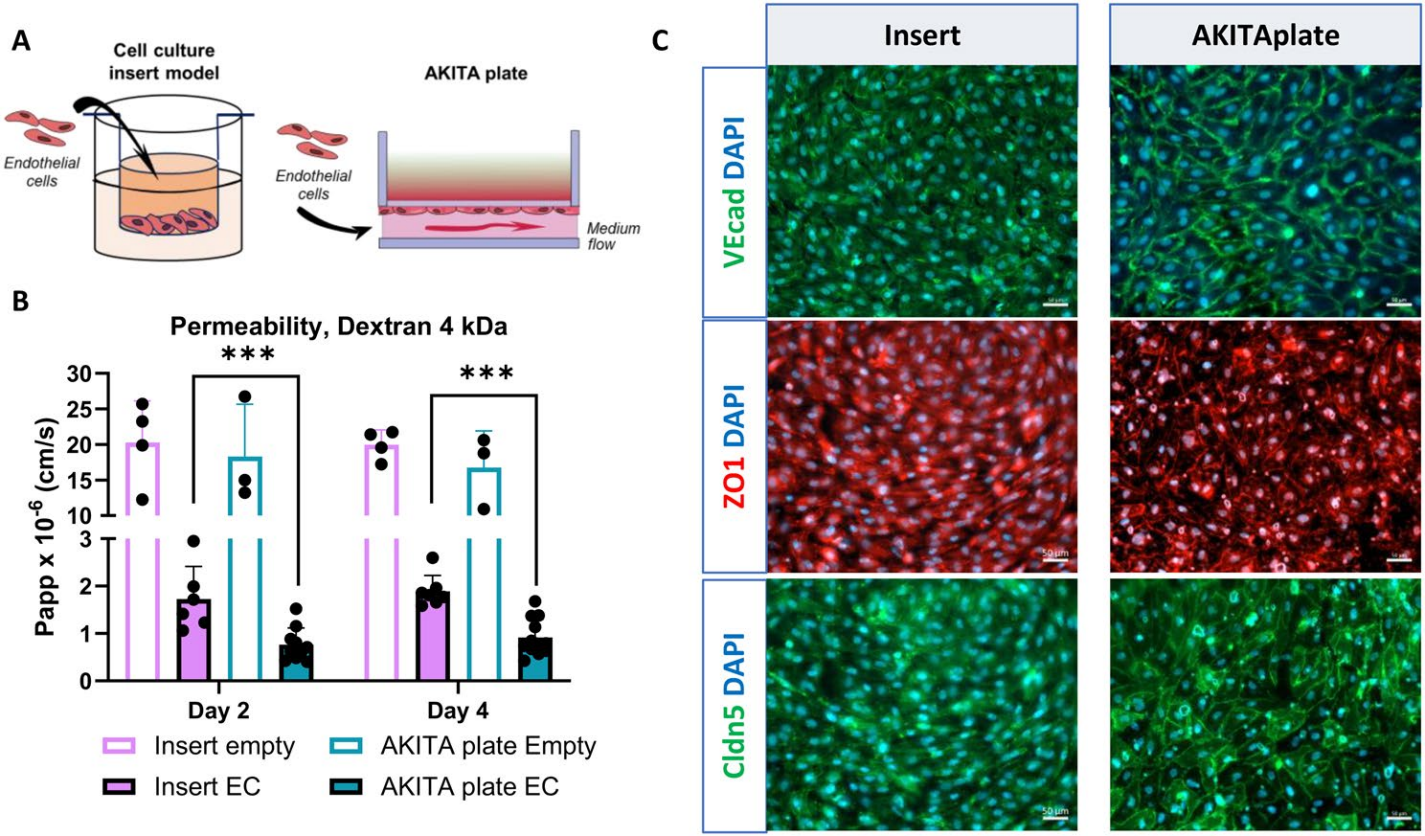
Enhanced barrier function of hiPSC-ECs on AKITA plate (**A**) Illustration of a culture setup. ECs were cultured on cell culture insert or AKITA plate for 4 days. (**B**) Permeability (Papp) values of 4 kDa Dextran on days 2 and 4 from ECs cultured on inserts or AKITA plate. *n* = 3–12, two independent experiments (empty), three independent experiments (cells). Two-way ANOVA with Šídák´s multiple comparison test, ***p < 0.001. (**C**) Representative immunofluorescence images of ECs cultured on inserts or AKITA plate stained with VE cadherin (VEcad), claudin 5 (Cldn5), and ZO1. Nuclei stained with DAPI. Scale bar 50 μm. ZO1; Zonula Occludens-1. All data presented as mean +/-SD

Next, we evaluated the impact of the cell culture model on ECs function by comparing ECs cultured on an AKITA plate with those of a traditional cell culture insert model (Fig. 2A). First, we assessed the barrier tightness with 4 kDa Dextran in a four-day culture. ECs grown on AKITA plate had significantly lower permeability in comparison with those cultured on inserts (Fig. 2B, Fig S2A). No significant differences were detected between the empty inserts and the AKITA plate. Additionally, decreased permeability for LY on day 4 was observed for ECs grown on AKITA plate, however, the change was not significant (Fig S2B). Further analysis of Ve cadherin, claudin 5 and ZO1 protein expression showed more robust expression in ECs cultured on the AKITA plate (Fig. 2C). We additionally noticed that ECs cultured on inserts proliferated on both sides of the membrane, a phenomenon not seen with ECs on the AKITA plate (Fig. S2C).

### The transcriptome of hiPSC-endothelial cells is affected by the culture model

**Fig. 3:**
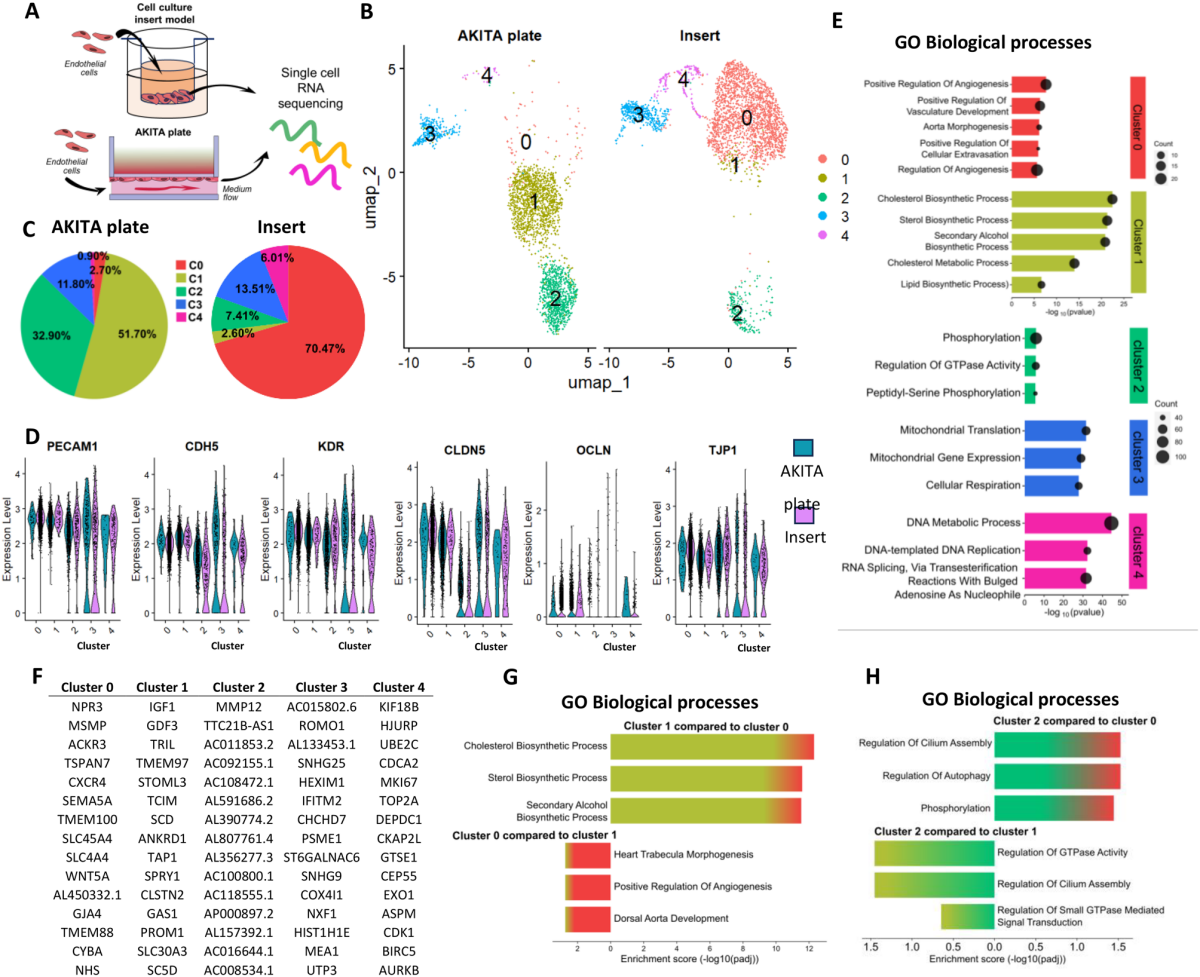
A single-cell RNA sequencing analysis of ECs cultured on the AKITA plate or inserts shows a distinct transcriptional difference. (**A**) Illustration of the study design. ECs were cultured on cell culture inserts or AKITA plate for 5 days before cell collection. (**B**) Uniform Manifold Approximation and Projection (UMAP) plot displaying clusters in ECs cultured on inserts or AKITA plate. Different colors indicate distinct cell clusters; each dot represents one cell. *n* = 3424 cells (insert), 3037 cells (AKITA plate). (**C**) Pie chart showing the clustering of the cells as percentages in ECs cultured on inserts or AKITA plate. (**D**) Violin plots depicting the expression of EC genes *EPCAM*, *CDH5*, and *KDR* and tight junction genes *CLDN5*, *OCLN*, and *TJP1* in ECs grown on inserts or AKITA plate. (**E**) Top GO biological processes derived from DEGs enriched in each cluster. (**F**) Top 15 genes in each cluster based on log2Fold change. (**G**) Top GO biological processes from DEGs between clusters 1 and 0. (**H**) Top GO biological processes from DEGs in cluster 2 when compared to clusters 0 or 1

Single-cell RNA sequencing (scRNA sequencing) was performed to investigate the differences between ECs cultured on AKITA plate and inserts (Fig. 3 A). We noticed a clear difference in cell clustering across different culture systems (Fig. 3B, C). In ECs cultured on inserts, the majority were located in cluster 0, whereas ECs cultured on AKITA plate resided in cluster 1. Differences were also observed in other clusters; cluster 2 was more prominent in AKITA plate ECs, and cluster 4 was more prevalent in insert ECs. Subsequently, we checked if the cells in different clusters were expressing EC-related genes (Fig. 3D, Fig S3A-D). Although most of the genes were expressed in cells in different clusters, there were some deviations. For example, tight junction protein *CLDN5*, cell-cell adhesion-related genes *ESAM* (endothelial cell adhesion molecule), and *S1PR1* (sphingosine-1-phosphate receptor 1) were less enriched in cluster (2) *ESAM*, vascular development-related gene *TEK* (TIE2), and *VWF* (von Willebrand factor), a highly selective endothelial marker participating in hemostasis, were less enriched in cluster 3. Overall, the expression of *OCLN* was lower in all clusters compared to the rest of the junction genes. GO biological processes (GO BP) analysis showed upregulation of vascular and angiogenesis-related pathways in cluster 0, whereas cholesterol and lipid-related pathways were enriched in cluster 1 (Fig. 3E). The genes enriched in cluster 2 were connected to phosphorylation. Genes in cluster 3 were associated with mitochondria and cellular respiration, while genes in cluster 4 pertained to DNA replication. Genes linked to these pathways were also identified among the leading genes specific to each cluster, including *SEMA5A* and *WNT5A* for angiogenesis, *SC5D* for cholesterol biosynthesis, *COX4I1* for cellular respiration, and *TOP2A*, *MKI67* and *DEPDC1* for DNA metabolic processes (Fig. 3F). Given that clusters 0 and 1 had the highest enrichment of ECs, we compared the enriched genes across these clusters using the find markers command and performed the pathway analysis. GO BP analysis yielded results consistent with prior findings, including cholesterol biosynthesis and angiogenesis (Fig. 3G). Since the top genes of cluster 2 showed several non-coding genes, we took a closer look at cluster 2 and examined the pathways linked to enriched genes when compared to cluster 0 or 1 (Fig. 3H). Besides phosphorylation and GTPase activity, regulation of cilium assembly was upregulated in this cluster.

### HiPSC-astrocytes require a 3D environment to maintain the endothelial cell barrier

**Fig. 4:**
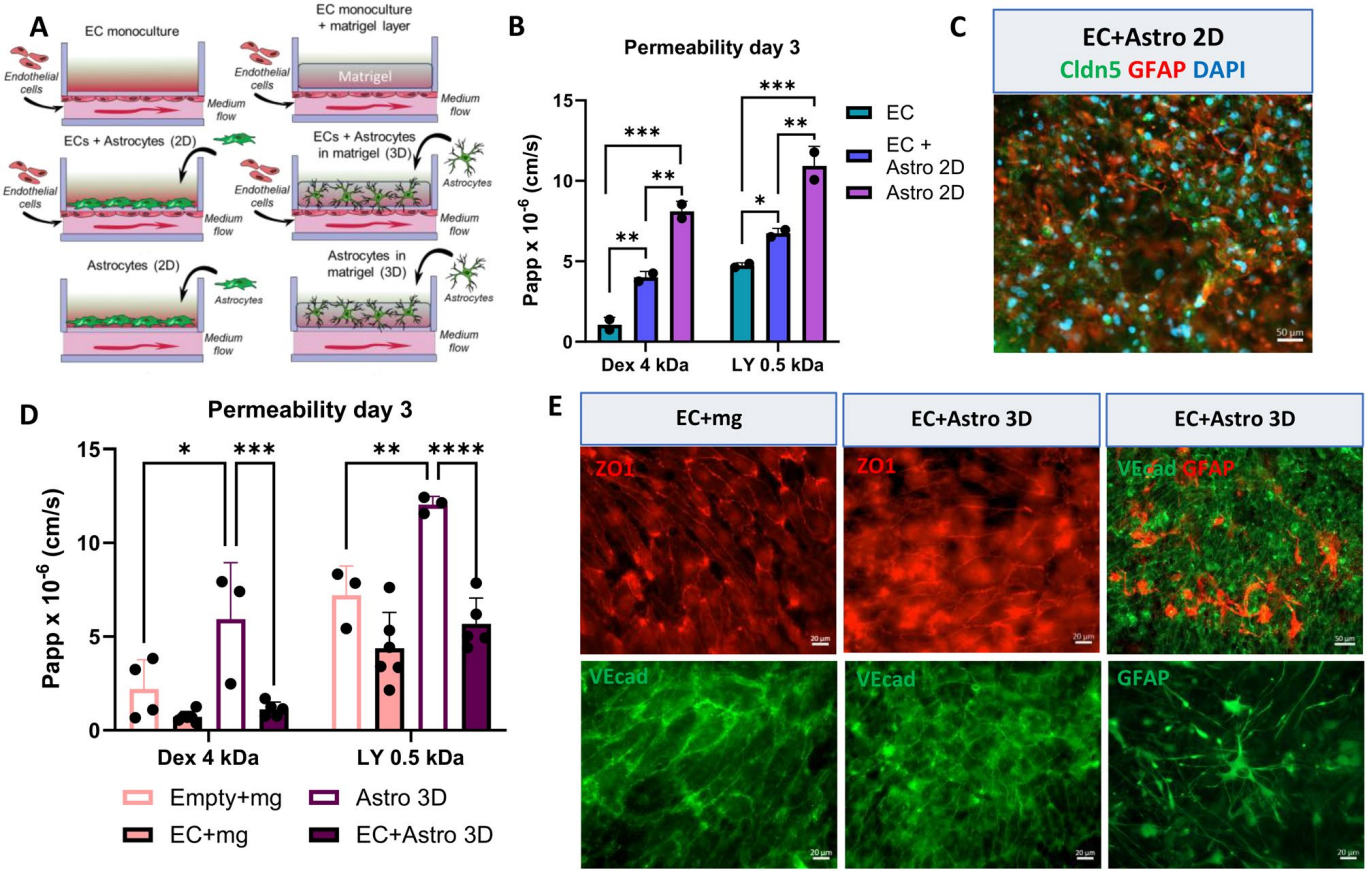
Astrocytes disrupt endothelial cell barrier in 2D culture but maintain it in 3D (**A**) Study design for the co-culture setup in the AKITA plate. (**B**) Permeability (Papp) values of 4 kDa Dextran and LY from EC monoculture, EC-astrocytes 2D culture, and astrocytes monoculture. *n*=2, Two-way Anova with Tukey´s multiple comparison test, *p<0.05, **p<0.01, ***p< 0.001. (**C**) Representative immunofluorescence staining of ECs (claudin 5, green) and astrocytes (GFAP, red) in 2D co-culture. Nuclei stained with DAPI (blue). Scale bar 50 μm. (**D**) Permeability (Papp) values of 4 kDa Dextran and LY from EC monoculture with Matrigel, empty well with Matrigel, EC-astrocytes 3D culture, and astrocytes in 3D alone. *n*=3-6, two independent experiments. Two-way Anova with Tukey´s multiple comparison test, *p<0.05, *p<0.01, ***p< 0.001. (**E**) Representative immunofluorescence images of ECs cultured on the AKITA plate with or without astrocytes in 3D. ECs stained with ZO1 (red) and VE-cadherin (green), astrocytes with GFAP. Scale bar 20–50 μm. Claudin 5, cldn5; VE cadherin, VEcad; Zonula Occludens-1, ZO1. All data presented as mean +/-SD

Considering that astrocytes maintain the BBB barrier function, we aimed to explore the impact of including astrocytes on the properties of ECs (Fig. 4A) when cultured on the AKITA plate. First, we plated astrocytes directly on the open top chamber with a thin layer of Matrigel coating to mimic 2D culture system. Subsequently, we measured permeability using 4 kDa Dextran and LY. Surprisingly, in 2D co-culture setup, the permeability was higher compared to ECs monoculture alone, suggesting a loss of barrier properties (Fig. 4B). Immunostainings showed a clear loss of ECs and infiltration of astrocytes, identified with claudin 5 and GFAP staining, respectively (Fig. 4C). To overcome the situation, we cultured the astrocytes in 3D conditions by using thicker Matrigel layer. This improved the permeability values (Fig. 4D). As controls, we used empty wells with Matrigel layers or only astrocytes in Matrigel. ECs co-cultured with astrocytes in 3D had significantly lower permeability compared to astrocytes cultured alone. We further confirmed that ECs grown on the AKITA plate, irrespective of the presence of astrocytes, expressed VE-cadherin and ZO1 (Fig. 4E). Astrocytes interacted with ECs and showed typical morphology when stained with GFAP.

### Astrocytes induce a subtle yet notable shift in a specific endothelial cell cluster

**Fig. 5:**
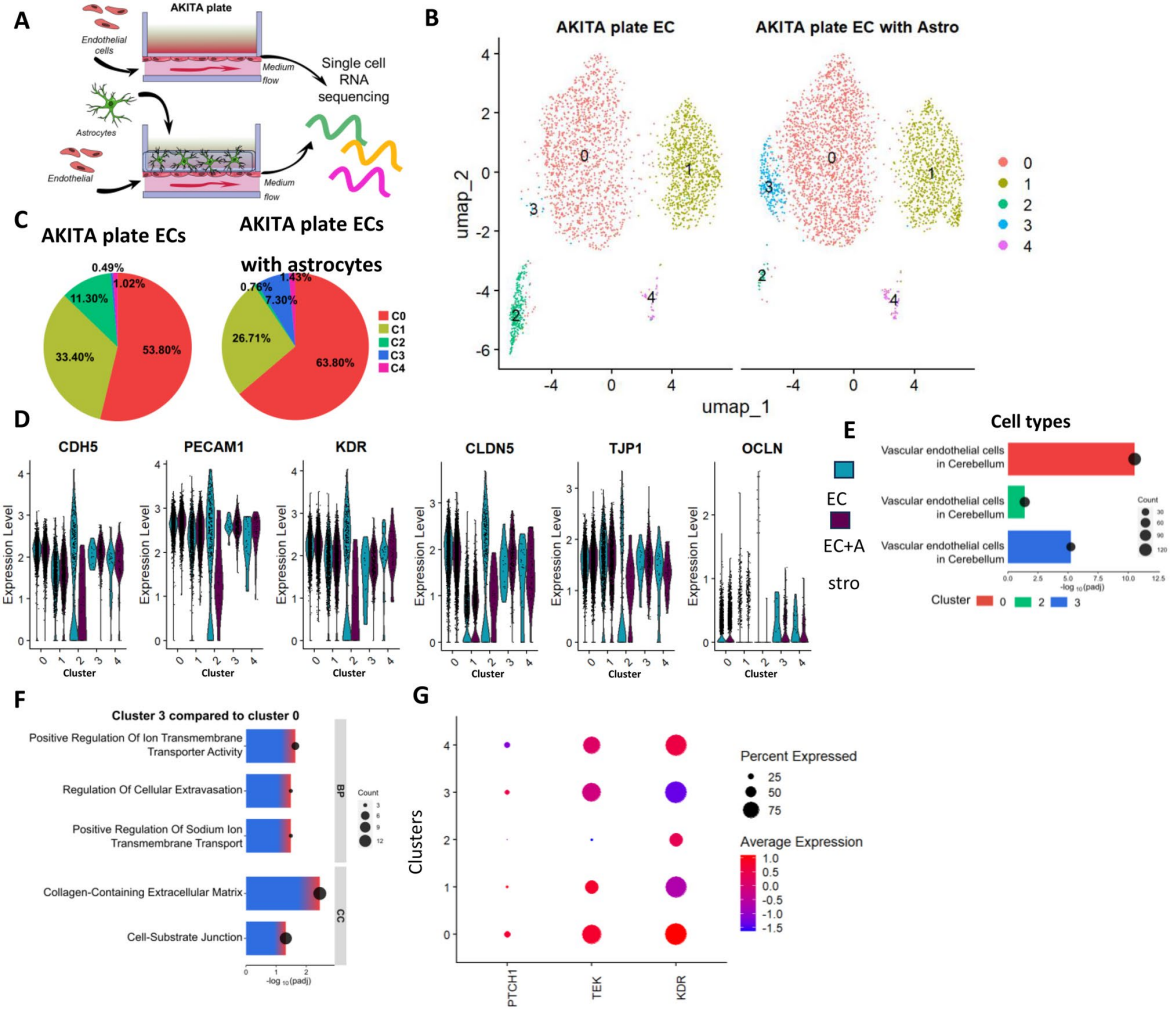
An scRNA sequencing analysis of ECs cultured on the AKITA plate with astrocytes induce a shift in one cluster. (**A**) Illustration of the study design. ECs were cultured on an AKITA plate with or without astrocytes for 4 days before the cells were collected. (**B**) Uniform Manifold Approximation and Projection (UMAP) plot showing clusters in ECs cultured on the AKITA plate with or without astrocytes. Different colors indicate distinct cell clusters; each dot represents one cell. *n*=3037 cells (AKITA plate, ECs), 3558 cells (AKITA plate, ECs+astrocytes). (**C**) Pie chart showing distribution of the cell clusters as percentages in ECs cultured with or without astrocytes on the AKITA plate. (**D**) Violin plots showing the expression of EC genes *EPCAM*, *CDH5*, and *KDR*, and tight junction genes *CLDN5*, *OCLN*, and *TJP1* expression in ECs cultured with or without astrocytes on the AKITA plate. (**E**) Most enriched cell type based on DEGs from clusters 0, 2, and 3. (**F**). Top GO biological processes (BP) and Cellular components (CC) from DEGs in cluster 3 when compared to cluster 0. (**G**) Dot Plot showing expression of *PTCH1*,* TEK*, and *KDR* in each cluster

To investigate the impact of astrocytes on the ECs transcriptome when cultured on the AKITA plate, we performed scRNA sequencing (Fig. 5A). The main differences were seen in clusters 2 and 3 (Fig. 5B, C). Cluster 2 was enriched in ECs devoid of astrocytes, whereas cluster 3 was enriched in ECs co-cultured with astrocytes. Additionally, the presence of astrocytes led to an increased proportion of cells in cluster 0. We re-evaluated the expression of EC-related genes and found similar trends as previously (Fig. 5D, Fig. S4A). Cluster 1 had lower expression of *CLDN5*, *ESAM*, and *S1RP1*, whereas cluster 2 had *VWF*, *ESAM*, *TEK*, and *S1RP1*. In contrast, gene expression profiles in cluster 3 resembled those in cluster 0, whereas cells in cluster 2 had a lower percentage of cells expressing these genes. Furthermore, we analyzed the expression of transporters, receptors, and metabolizing enzymes associated with BBB (Fig. S4B-D). ECs did not express all these genes, but several genes were more expressed in cluster 3 compared to cluster 2. Focusing on clusters 0, 2, and 3, we identified the cell types based on the DEGs. The most enriched cell types linked to these clusters were vascular endothelial cells in the cerebellum (Fig. 5E). Further analysis of cluster 3 revealed distinct gene expression patterns when compared to cluster 0. Pathway analysis of biological processes indicated that genes upregulated in cluster 3 were associated with ion transmembrane transporters and cellular extravasation (Fig. 5F). Cellular components showed enrichment of genes linked to collagen-containing extracellular matrix and cell-substrate junction. Finally, we examined the expression of key receptors linked to EC-astrocyte communication inside each cluster (Fig. 5G). The percentage of cells expressing *PTCH1*, *TEK* (Tie-2) and *KDR* (VEGFR2) increased in cluster 3 compared to cluster 2. The mean expression levels of *PTCH1* and *TEK* were also elevated in cluster 3. However, *KDR* expression was reduced in comparison to cluster 2.

## Discussion

The proper function of the BBB maintains a healthy brain environment. Reliable human in vitro BBB models are needed for drug and disease studies. Microfluidic organ-on-chip models have become increasingly accessible and have gained interest in the field of BBB modeling. However, considerable variation persists in the used models and cell types. Understanding the impact of various culture systems on cellular function might yield enhanced insights into these models and, as a result, ultimately resulting in improved models in the future.

Typically, BBB chip models consist of two channels separated by a porous membrane. Previous studies with hiPSC-ECs on BBB chips with membranes have used PET, PC, or PDMS membranes with pore sizes ranging from 0.4 to 8 μm [7]. However, the investigation of how different pore sizes affect ECs’ function has been minimal in these studies. Motallebnejad et al. demonstrated that ECs form a confluent layer in 0.4 and 8 μm sized membranes; still, for 8 μm membranes, the presence of hydrogel on the opposite side was essential to prevent cell migration [25]. Moreover, previous studies with HUVECs have shown that a smaller pore size can enhance tight junction formation, cell growth, and attachment [26]. In our model, a confluent monolayer was observed across all studied pore sizes, but interestingly, for a 1 μm-sized membrane, the formation was notably prolonged. Furthermore, we did not detect cell migration even with higher pore sizes. Altogether, the AKITA plate model supports the formation of EC monolayer even with larger pore sizes.

One of the main aims of the study was to explore the impact of different culture setups on the barrier function and transcription of hiPSC-ECs. We noticed a significant decrease in permeability of hiPSC-ECs cultured on the AKITA plate compared to the insert model, with no significant differences among the empty units. This was not supported by the scRNA sequencing data, which did not show any considerable upregulation of tight junction genes in ECs cultured on the AKITA plate. However, we observed a more robust protein expression of VE cadherin, ZO1 and claudin 5 in ECs cultured on the AKITA plate. The effect of flow and shear stress has been examined in human brain microvascular ECs as well as ECs from rats, bovines, and pigs. Although these studies reported increased TEER, lower permeability, and upregulation of tight junctions [27], studies involving hiPSC-derived ECs and hCMEC/D3 line did not show any increase in the expression levels of junctional proteins [28, 29]. In a study with hiPSC microvascular ECs, DeStefano et al. argued that flow induces an increase in contact area, hence strengthening the barrier function; however, as the tight junctions are already formed under static conditions, this does not lead to the overexpression of tight junction proteins or genes [28]. Likewise, brain-like ECs derived from CD34 + hematopoietic stem cells showed marked improvement in barrier function under flow, but no upregulation of tight junctions [30]. Taken together, the effect of flow on BBB-related markers can be dependent on the cell type and the models used. There is still need for further studies to better understand the underlying mechanisms of how flow alters EC function and barrier properties.

One interesting finding was that when hiPSC-ECs were cultured on inserts, cell migration to the other side of the membrane was evident. This was not seen when cells were cultured on the AKITA plate. The scRNA sequencing data confirmed this, indicating a greater number of proliferating cells in hiPSC-ECs on inserts. Moreover, the genes related to the most prominent cluster in insert ECs were linked to angiogenesis, vascular development and cellular extravasation. This is in line with previous studies, which have shown decreased levels of proliferation, downregulation of cell motility and proliferation-related proteins, and inhibition of the cell cycle under shear stress [28, 29, 31, 32]. The reduced migration and proliferation of hiPSC-ECs on the AKITA plate may lead to enhanced barrier function.

Most enriched clusters in hiPSC-ECs cultured on the AKITA plates were 1 and 2. Upon investigation of the pathways related to cluster 1, cholesterol biosynthesis was upregulated. The brain is a cholesterol-rich organ, with astrocytes and oligodendrocytes producing cholesterol in situ [33]. The BBB and the blood–cerebrospinal fluid barrier isolate the brain cholesterol from the blood circulation. However, a part of the cholesterol pool is exchanged between the brain and blood as metabolites. Although ECs are important in controlling the brain’s cholesterol levels, little is known about how endothelial cholesterol regulation affects brain function. A recent report by Profaci et al. showed that neuronal activity increases the expression of several cholesterol biosynthesis-related genes in brain ECs in vivo, suggesting that cholesterol biosynthesis may serve as a negative feedback loop for neurovascular coupling [34]. Interestingly, shear stress similarly upregulated cholesterol genes in hiPSC-brain EC-like cells in vitro, indicating that blood flow mediates the activity-dependent control of brain endothelial cholesterol metabolism. The increased cholesterol biosynthesis in ECs did not affect the brain cholesterol levels, suggesting a more local function in ECs. The second difference between ECs cultured on the AKITA plate and inserts was observed in cluster 2, which was more prominent in the AKITA plate ECs. This cluster contained several non-coding RNAs, yet a detailed examination revealed genes linked to phosphorylation and cilium assembly. Cilia are microtubule-based organelles present in several cells, including ECs [35]. The role of cilia in ECs has been linked to sensing fluid shear stress and transmitting signals [36–38]. In addition, studies have shown that cilia contribute to the development and maintenance of vascular stability [39–42]. Collectively, these findings indicate that flow or shear stress can induce transcriptional changes beyond typical barrier-related genes. Furthermore, the results suggest that the regulation of vascular integrity and function is a complex phenomenon and understanding all the aspects requires more research.

Astrocytes are essential for the formation and maintenance of the BBB. When astrocytes were cultured in a 2D on the open-top chamber of the AKITA plate, it resulted in higher permeability compared to monoculture and infiltration of astrocytes to the vascular side. This was reversed when astrocytes were plated in 3D using a Matrigel layer. Matrigel likely restricts astrocytes movement, allowing ECs to form a confluent layer. Previous studies have also shown that astrocytes cultured in 2D exhibit a reactive phenotype [43, 44]. Thus, 3D culturing of astrocytes may reduce the reactivity and, therefore, support the EC barrier formation. Empty units with Matrigel layers had significantly lower permeability when compared to Matrigel layers with astrocytes. This indicates that astrocytes enhance the permeability of the Matrigel layer, rendering the comparison between monoculture and 3D co-culture impractical. A significant decrease in permeability was observed when comparing the EC-astrocyte co-culture to empty units with astrocytes. This was not seen with monoculture when compared to empty units with only Matrigel layers. This points to better barrier formation in co-culture conditions. Several studies with different types of in vitro models have reported improved barrier function, including increased TEER values or lower permeability, especially with primary and immortalized cell lines [45]. However, hiPSC-derived ECs co-cultured with astrocytes alone don´t always demonstrate marked improvement of the barrier function [7]. In some studies, improved barrier function has been observed only with co-culture with several cell types [46, 47]. In sum, astrocytes are known to enhance and maintain the barrier function in ECs, but this does not always result in decreased permeability values, especially in case of iPSC-derived ECs.

ECs cultured with or without astrocytes showed minor differences in transcription level. A shift in one cluster was noticed when ECs were cultured with astrocytes. Furthermore, there is an increased number of cells in cluster 0 in ECs cultured with astrocytes. Analysis of cell types showed that genes from these clusters were associated with vascular endothelial cells in the cerebellum, with cluster 0 exhibiting a higher gene count and significance. This may suggest a transcriptional shift towards more BBB-like ECs when cultured with astrocytes. Gene expression analysis of BBB associated genes supported this, as an increased expression of many of these genes was detected in astrocyte specific cluster 3 compared to cluster 2. An in-depth pathway analysis of cluster 3 revealed an enrichment of ion transport activity. The ion balance in the brain is carefully controlled, and the selective transport of ions across the BBB sustains this balance [48]. Astrocytes may influence ion transport regulation in brain ECs. Astrocytes in the brain release several factors that support BBB integrity, including Sonic Hedgehog and Angiopoetin-1 [3]. Sonic Hedgehog interacts with the transmembrane receptor PTCH-1, and the activation of this pathway induces the expression of junctional proteins and promotes the BBB phenotype [49]. Angiopoetin-1 binds to Tie-2 receptor in ECs and promotes BBB integrity through junctional protein expression [50]. *PTCH1* and *TEK* (Tie-2) expression levels were upregulated in cluster 3 in comparison to cluster 2. The expression levels and percentages of cells expressing these genes were similar to those in cluster 0. In contrast to factors that sustain BBB, astrocyte-derived VEGF has a permeability-increasing effect, especially in neurological disorders [51]. Despite an increase in the number of cells expressing VEGFR2 receptor in cluster 3, the average expression level decreased compared to clusters 2 and 0. Astrocytes collectively generate modifications in a small subset of ECs via altering gene expression levels of receptors crucial for astrocyte-EC communication.

In summary, the culture system significantly influences the transcriptome of ECs. While improved barrier integrity was observed in ECs cultured on the AKITA plate, the transcriptomic differences were not primarily related to standard BBB-linked genes like junctional or transporter genes. This suggests that barrier integrity is a complex phenomenon supported by multiple factors and is not always directly reflected in the increased expression of tight junction genes. Also, the culture system alone did not induce the expression of all BBB related genes. Low expression of transporters, especially ABC transporters, has been reported in hiPSC-derived ECs [52]. Improvements in differentiation protocols are still needed for enhancing the brain like phenotype in hiPSC-ECs. For the transcriptomics, we only used one time point. A more comprehensive approach involving multiple time points could offer deeper insights into the dynamic nature of transcriptomic changes during EC maturation and BBB formation. Also, the addition of astrocytes alone had a minimal effect on the ECs transcriptome. This aligns with our understanding of BBB development, where ECs gradually mature to gain the specific features of brain ECs [53]. This is heavily guided by the surrounding cells, including pericytes and astrocytes. The recruitment of pericytes is critical for the formation of the BBB since they guide astrocytes toward the developing BBB [4]. It is also possible that the juvenile phenotype of hiPSC-derived ECs makes them unresponsive to astrocytic cues. The limited impact of astrocytes alone on the EC phenotype suggests that a more complete model, including pericytes, may be necessary to induce the brain EC phenotype fully.

### Limitation of the study

This study analyzed the effect of the culture system on ECs using transcriptomics and permeability assays. While these approaches provided valuable insights, additional functional tests, such as various transporter assays, would be necessary to better understand how the culture environment influences EC behavior. We compared the standard insert model to the chip, and although the biggest difference between these models is the addition of flow, we cannot conclusively attribute the observed effects to shear stress. It´s possible that the differences between the physical layouts of the model also contributed to the outcome. A more precise evaluation of shear stress could have been achieved by comparing ECs cultured on AKITA plates with and without flow. However, including inserts in the study was essential, as they are widely used in research and provide results for many users. For single-cell RNA sequencing, we compared ECs cultured with astrocytes in a 3D environment and EC monoculture without the additional Matrigel layer. It is possible that the Matrigel itself influenced the result. Ideally, a third group of ECs cultured with Matrigel layer should have been included for a more robust analysis. However, incorporating this additional group would have substantially increased the materials and resource requirements for the study. This study only included one hiPSC line, and it is important to keep in mind that these results could differ with different hiPSC lines.

## Conclusions

In conclusion, our results highlight the importance of considering culture conditions when studying EC behavior and barrier function. The AKITA plates, especially when combined with flow conditions, offer a promising platform for more physiologically relevant in vitro models of the BBB. Furthermore, the inclusion of astrocytes in a 3D environment appears to be crucial for maintaining EC monolayer integrity and promoting cell-cell interactions that may be important for barrier function. Future improvements are still needed to achieve more brain like hiPSC-ECs that are suitable for both drug and disease related studies.

## Electronic supplementary material

Below is the link to the electronic supplementary material.

Supplementary Material 1

## Data Availability

The scRNA sequencing data is available in the ZENODO repository. Inserts: 10.5281/zenodo.14892284, 10.5281/zenodo.15462020; AKITA plate EC: 10.5281/zenodo.15462033, 10.5281/zenodo.15462039; AKITA plate EC with astro: 10.5281/zenodo.15462058, 10.5281/zenodo.15462065.

## Abbreviations

BBB: Blood-brain barrier
DEG: Differentally expressed genes
EC: Endothelial cell
GO BP: Gene Ontology Biological Prosessess
hiPSC: Human induced pluripotent stem cell
LY: Lucifer yellow
Papp: Aapperent permeability coefficient
Pe: Permeability coefficient
PET: Polyethylene terephthalate
TEER: Transendothelial electrical resistance
