# Supplementary material for: From inserts to chips: microfluidic culture and 3D astrocyte co-culture drive functional and transcriptomic changes in hiPSC-derived endothelial cells
Source: Fluids Barriers CNS. 2025 Jun 16;22:58. doi: 10.1186/s12987-025-00672-7 (PMC12168306; doi:10.1186/s12987-025-00672-7)
Supplement: Supplementary file 1 — Supplementary Material 1 [file 12987_2025_672_MOESM1_ESM.docx]

# Supplementary information **Supplementary figure S1.** Representative immunofluorescence images of ECs cultured on AKITA plate with different pore sizes. ECs stained with CD31. Nuclei stained with DAPI. Images taken from both sides of the membrane. Scale bar 20 µm.


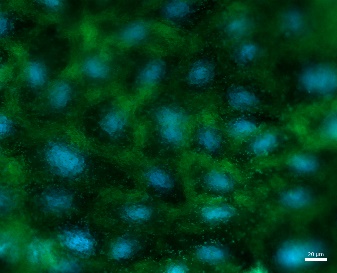

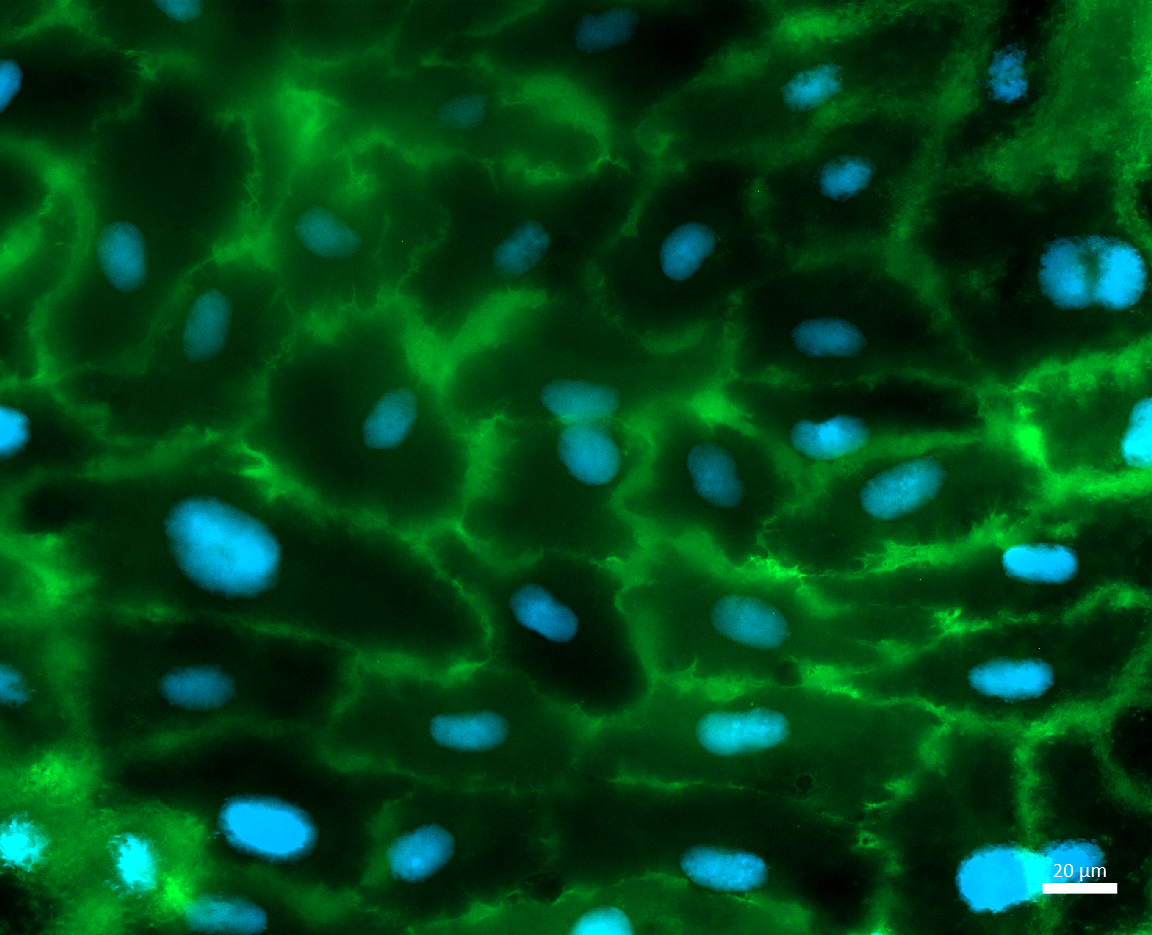

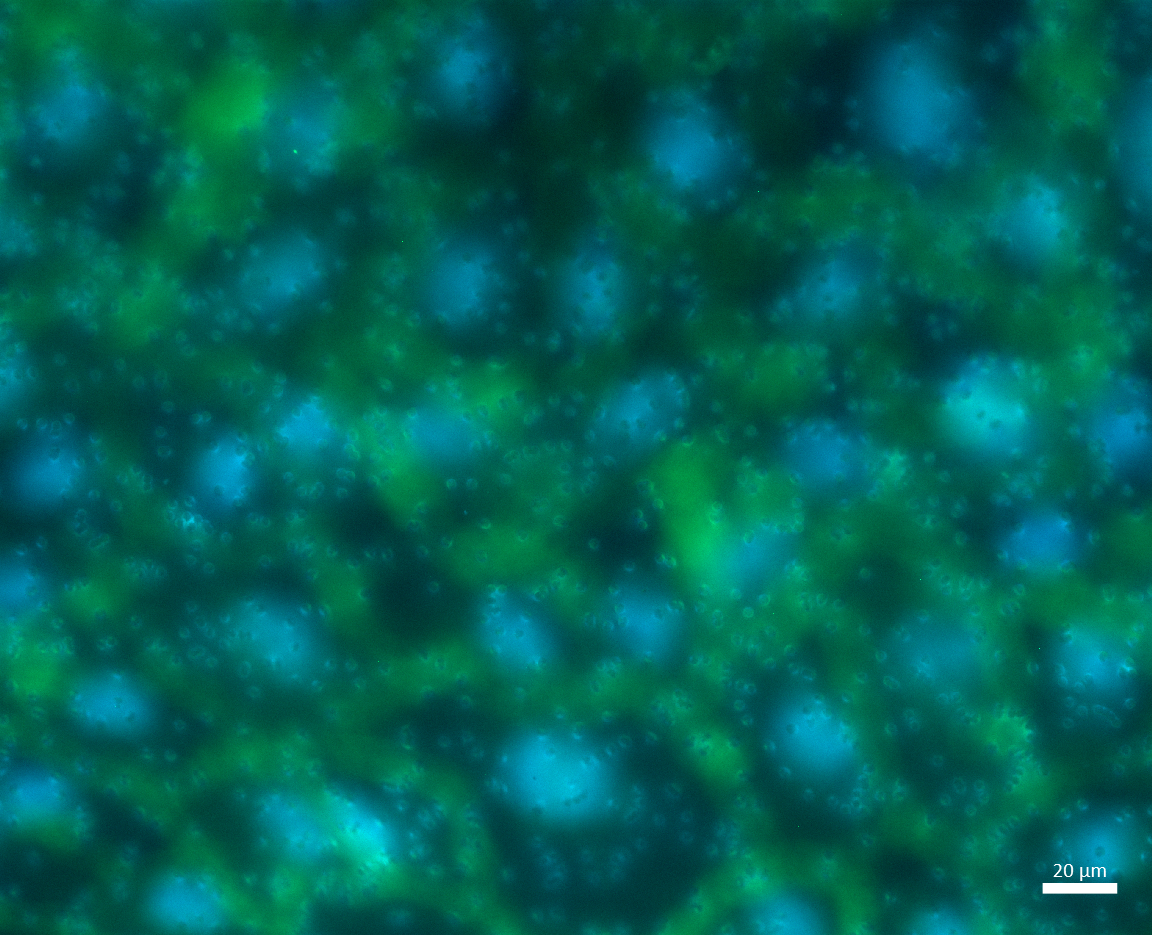

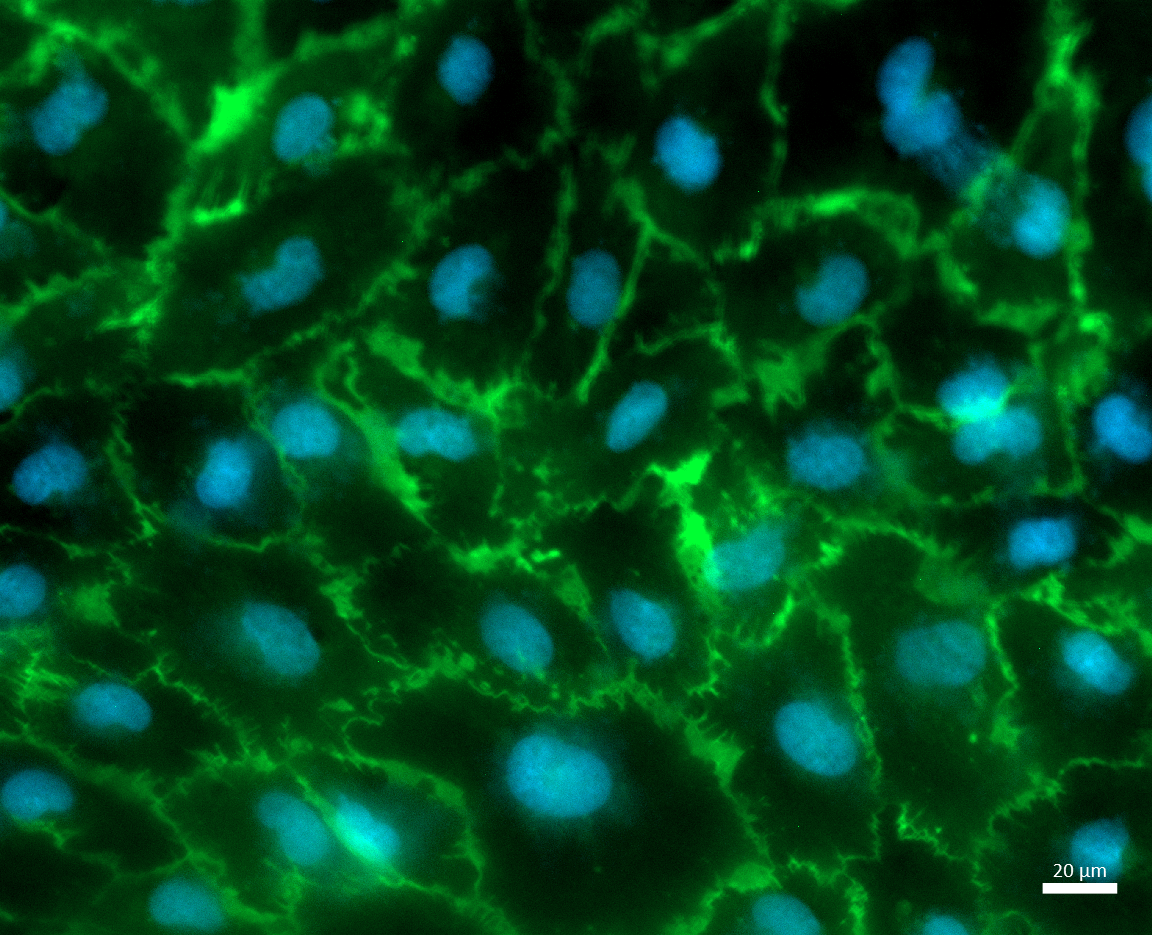

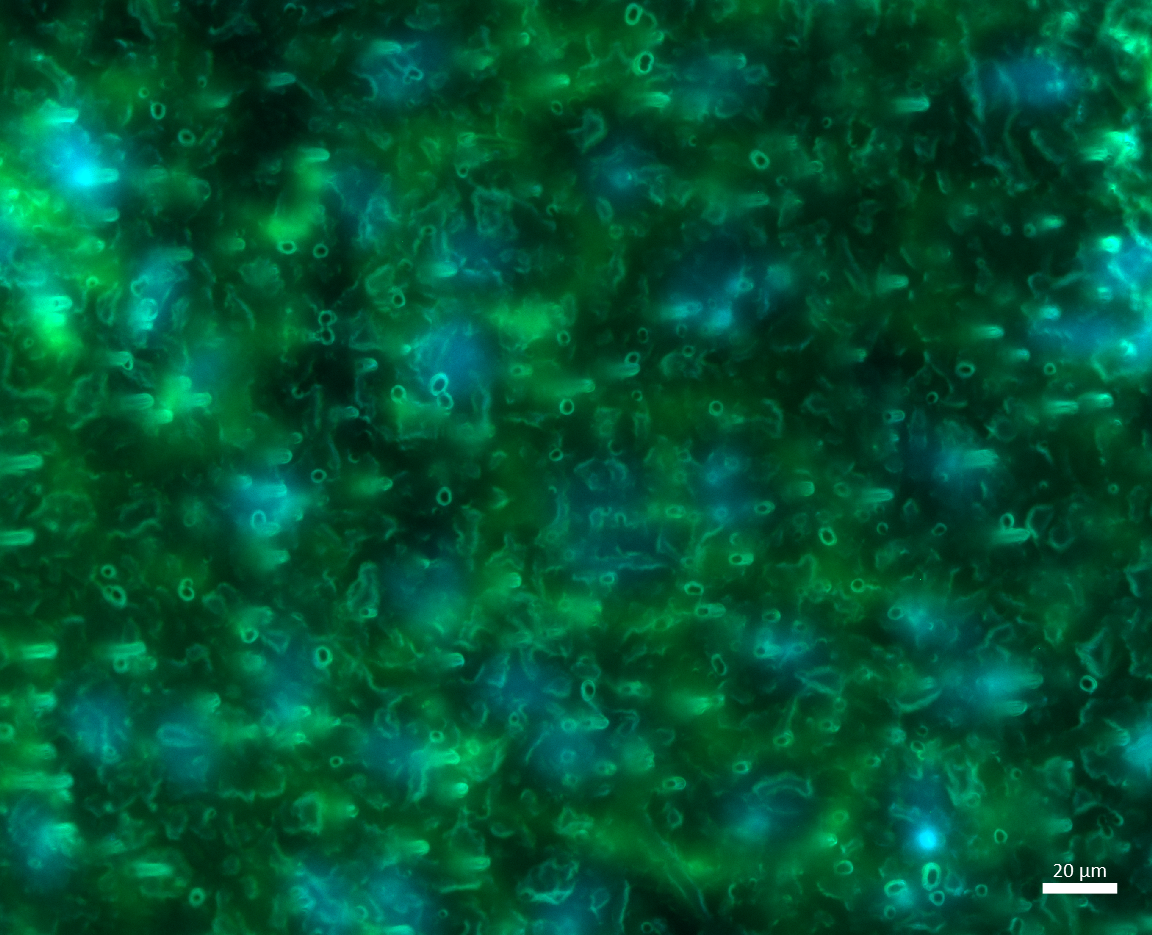

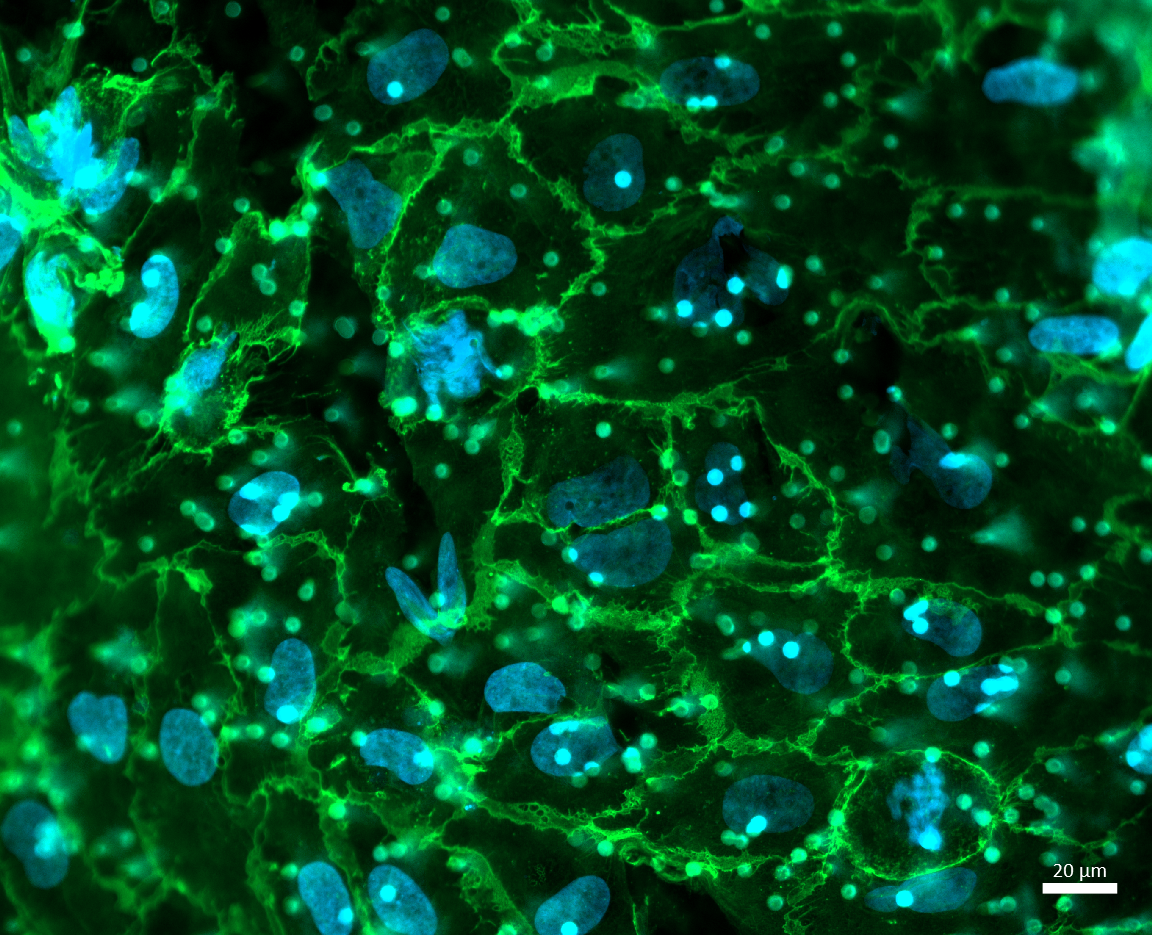

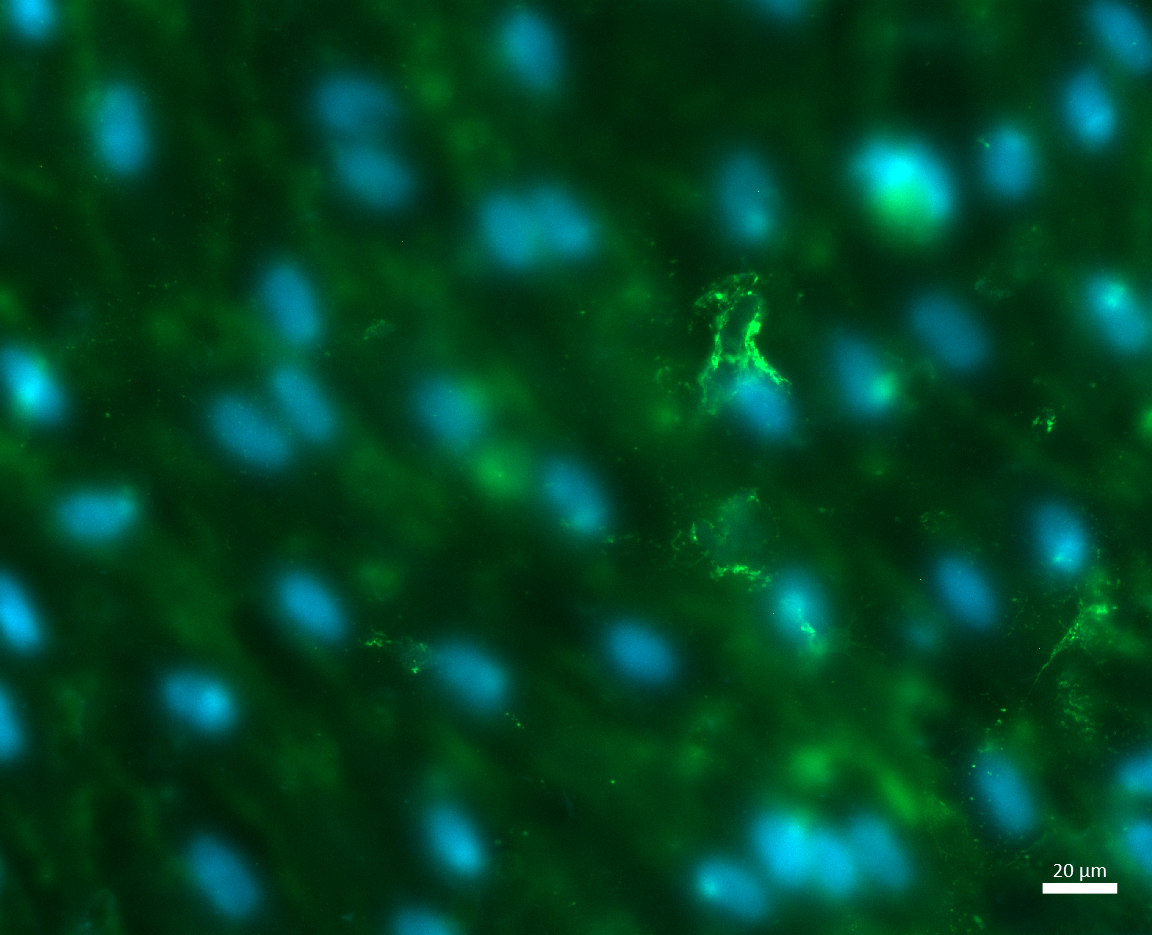

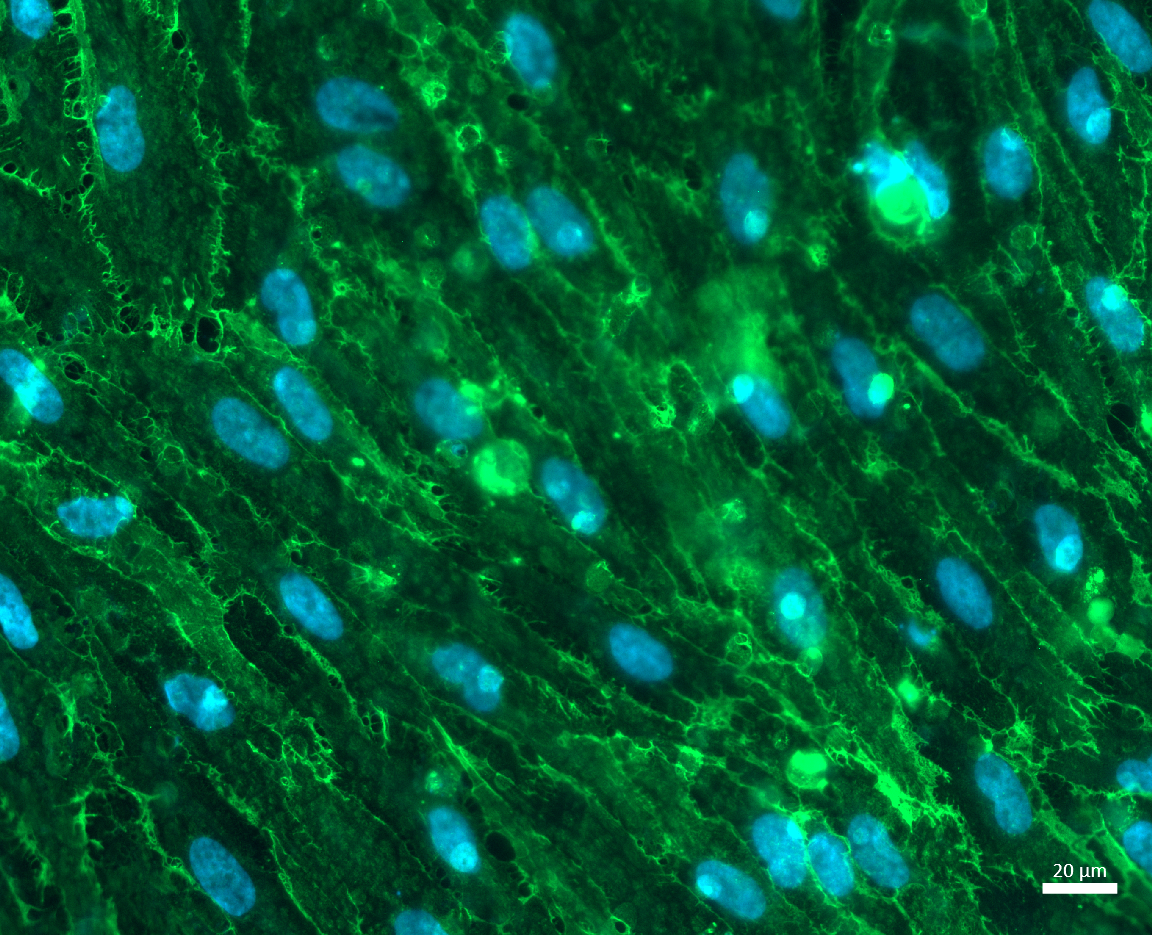


Membrane bottom (culture side)

Membrane

Top

**1 µm**

**3 µm**

**5 µm**

**8 µm**

**CD31 DAPI**

**CD31 DAPI**

**CD31 DAPI**

**CD31 DAPI**

Membrane

top (culture side)

Membrane

bottom


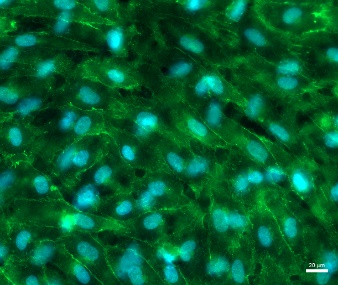


**Insert**

**VEcad DAPI**


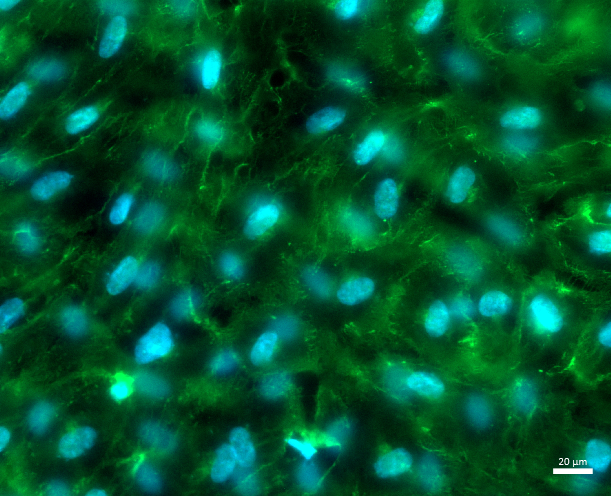


**VEcad DAPI**


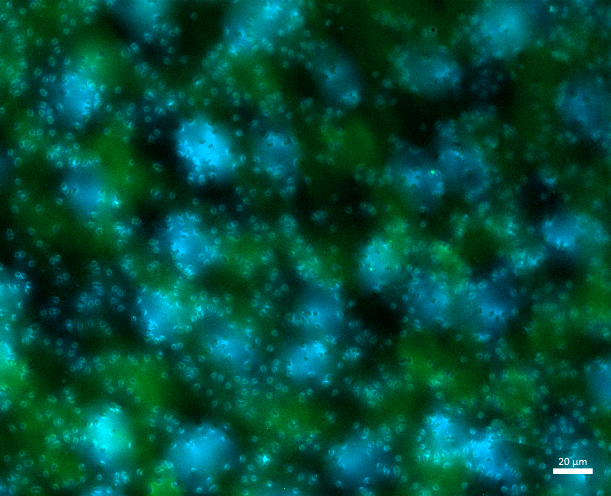

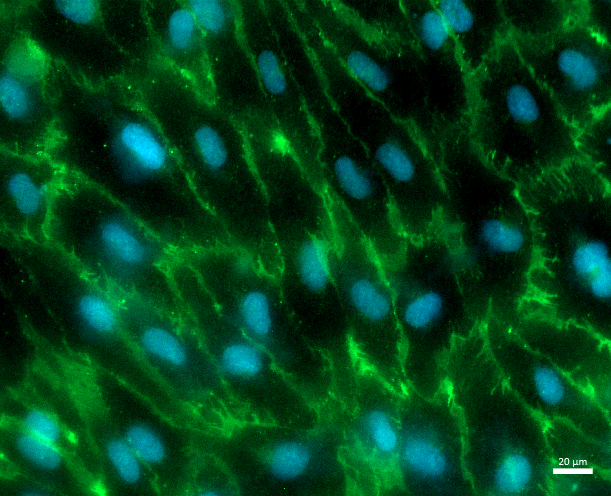


Membrane bottom (culture side)

Membrane

top

**VEcad DAPI**

**VEcad DAPI**

**AKITA pate**

**A**

**B**

**C**

**Supplementary figure S2.** (A) Permeability (Pe) values of 4 kDa Dextran on days 2 and 4 from ECs cultured on inserts or AKITA plate. n=2-4, two independent experiments. Two-way ANOVA with Šídák´s multiple comparison test, *p<0.05, *p<0.01. (B) Permeability (Papp) values of LY on day 4 from ECs cultured on inserts or AKITA plate. n=2-5, one independent experiment. One-way ANOVA with Tukey´s multiple comparison test, *p<0.05, **p<0.01 (C) Representative immunofluorescence images of ECs cultured on insert and AKITA plate with 3 µm pore size. ECs stained with VE cadherin (VEcad). Nuclei stained with DAPI. Images taken from both sides of the membrane. Scale bar 20 µm.


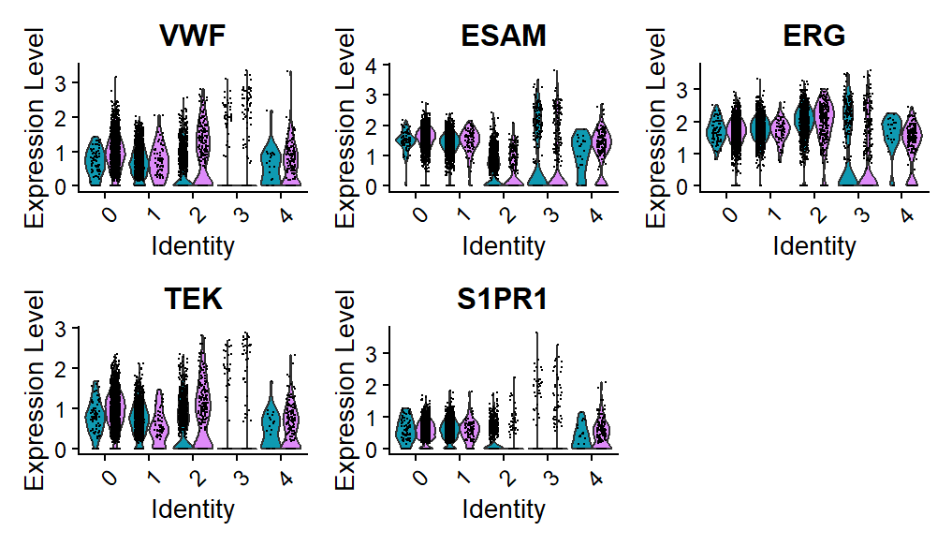

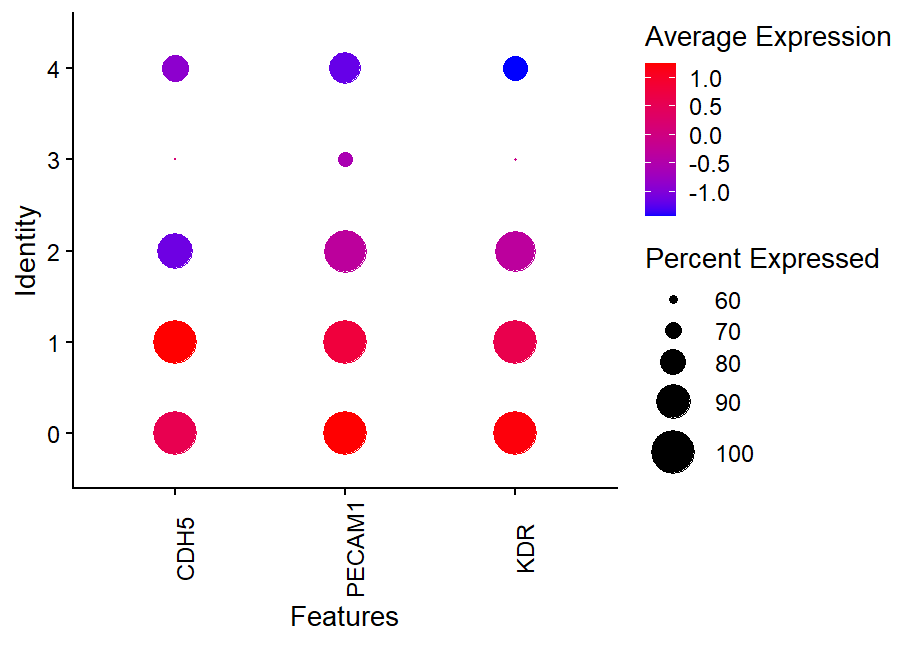

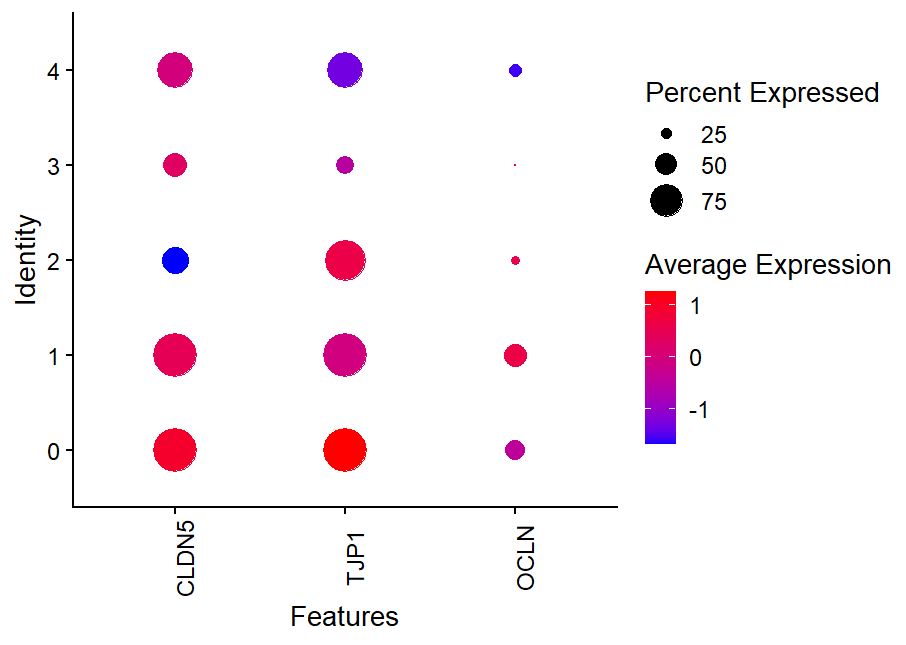

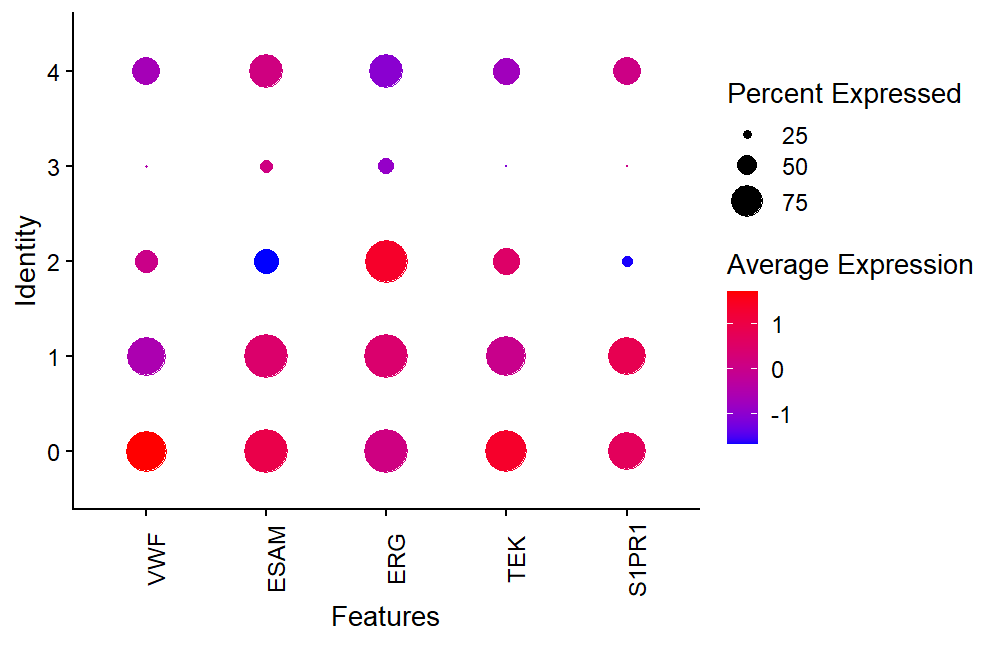


AKITA plate

Insert

Cluster

Cluster

Cluster

**A**

**B**

**C**

**D**

Cluster

Cluster

Cluster

Cluster

Cluster

**Supplementary figure S3.** Expression of EC related genes in ECs culture on insert or AKITA plate (A) Dot Plot showing average expression and percent of cells expressing EC genes *EPCAM*, *CDH5* and *KDR* and (B) tight junction genes *CLDN5*, *OCLN* and *TJP1* in each cluster. (C) Violin plot and Dot Plot (D) showing expression of EC genes *VWF, ESAM, ERG, TEK* and *S1PR1* in ECs cultured on inserts or AKITA plate.


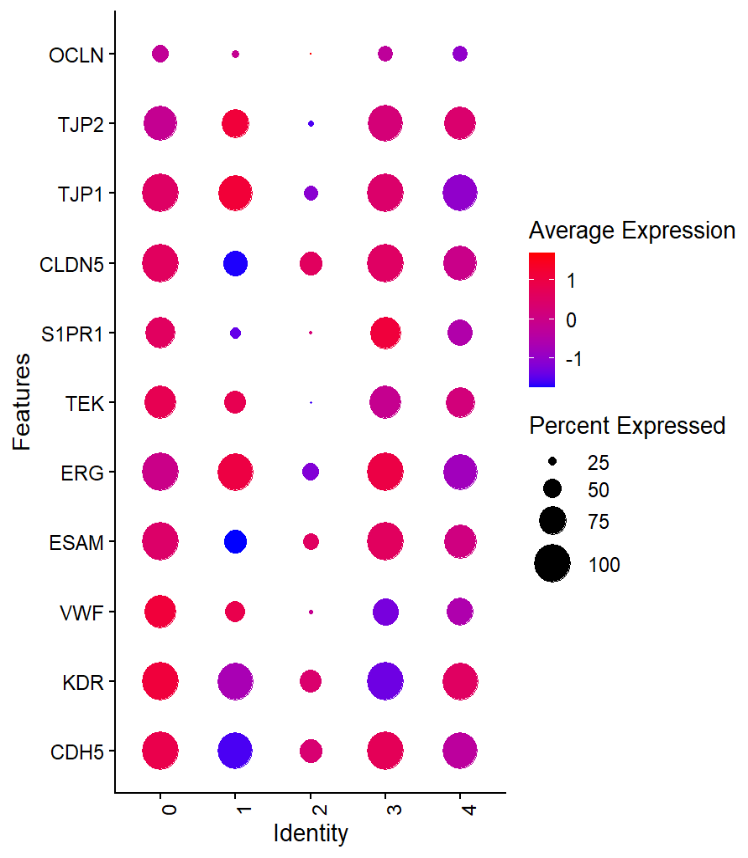


**A**

**C**

**B**


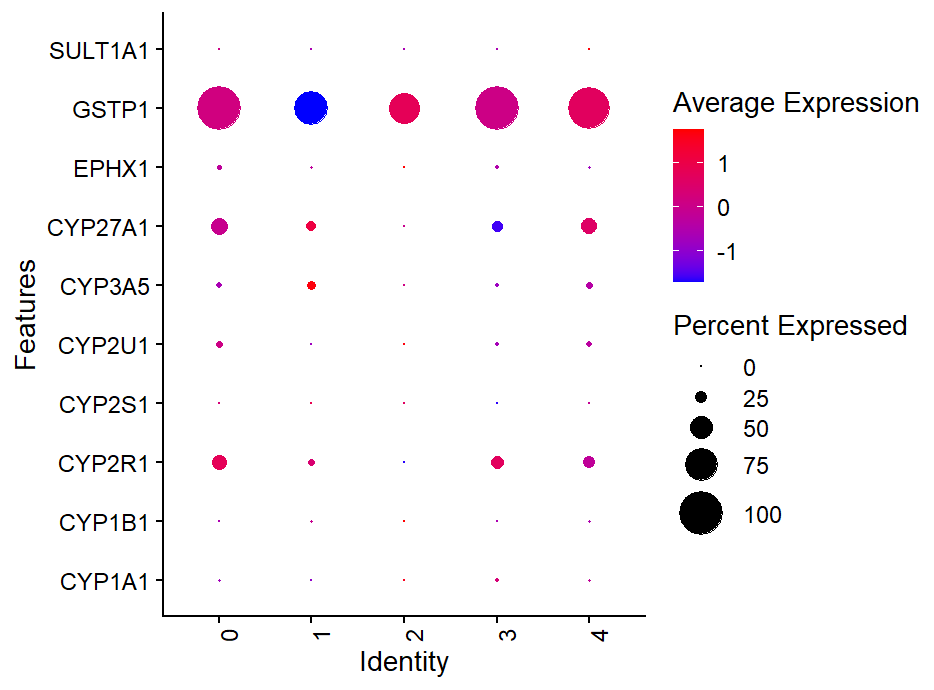


**D**


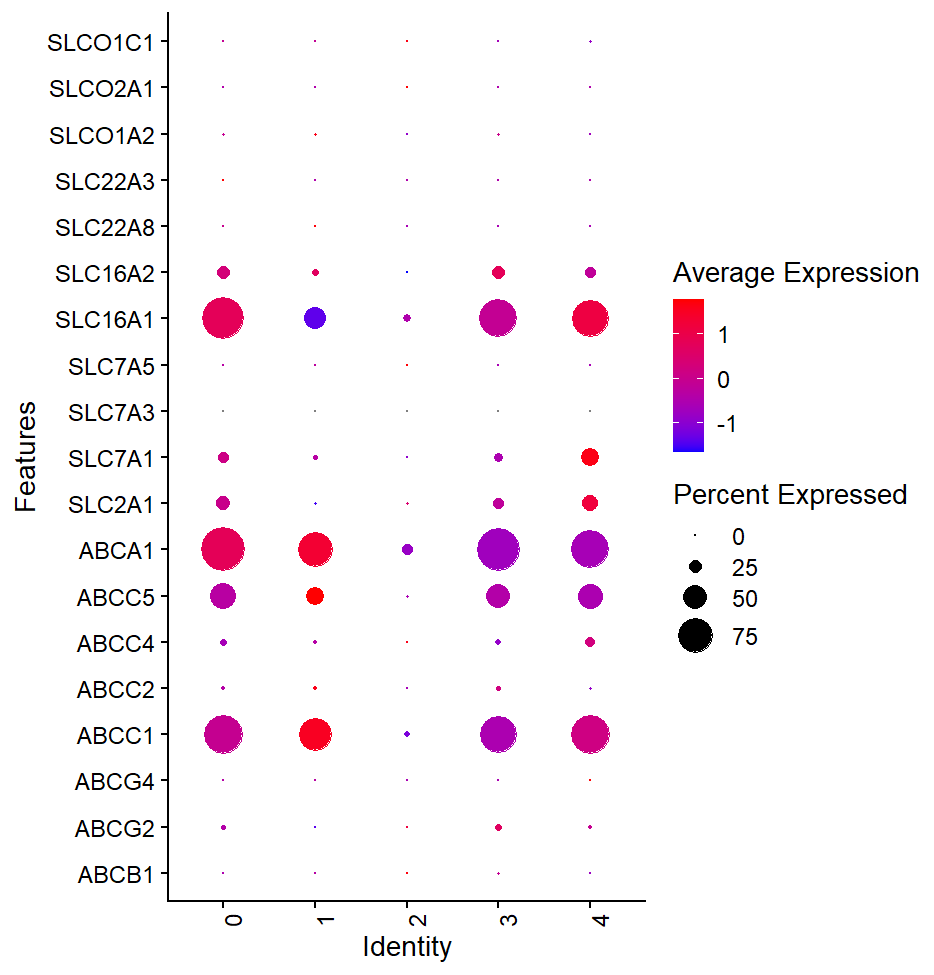

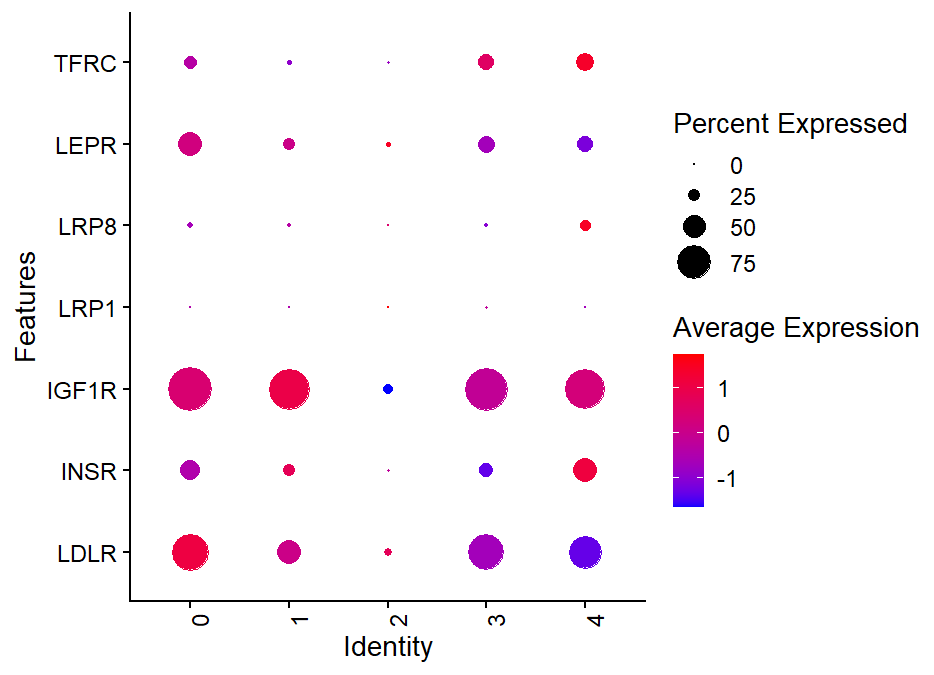


**Supplementary figure S4.** The expression of BBB related genes in ECs culture on AKITA plate with or without astrocytes. Dot Plot showing average expression and percent of cells expressing (A) basic EC genes and tight junctions (B) SLC and ABC transporters (C) receptors and (D) drug metabolizing enzymes.
